# Scenario analysis of nitrogen surplus typologies in Europe shows that a 20% fertilizer reduction may fall short of 2030 EU Green Deal goals

**DOI:** 10.1038/s43016-025-01210-2

**Published:** 2025-08-19

**Authors:** Masooma Batool, Fanny J. Sarrazin, Xin Zhang, Andreas Musolff, Tam V. Nguyen, Sabine Attinger, Rohini Kumar

**Affiliations:** 1 UFZ-Helmholtz Centre for Environmental Research, Leipzig, Germany; 2 Université Paris-Saclay, INRAE, UR HYCAR, Antony, France; 3 University of Maryland Center for Environmental Science, Frostburg, MD USA; 4 University of Potsdam, Potsdam, Germany

**Keywords:** Environmental impact, Element cycles

## Abstract

The Farm to Fork (F2F) Strategy under the Green Deal aims to halve nutrient losses by 2030 in the European Union (EU). Here, using the nitrogen surplus as an indicator for nitrogen losses in agricultural areas, we explore a range of scenarios for nitrogen surplus reduction across EU landscapes. We identify four nitrogen surplus typologies, each responding differently to input reduction. A 20% decrease in synthetic fertilizer alone is projected to reduce the nitrogen surplus by only 10–16%, falling short of F2F goals. Specific top-down scenarios such as reducing synthetic fertilizer by 43% and animal manure by 4%, coupled with improved technological and management practices, can achieve a reduction of up to 30–45% in nitrogen surplus. Among the most ambitious scenarios, only a handful of EU countries (four to five) may meet the intended F2F nitrogen pollution targets. Achieving F2F goals requires region-specific strategies to reduce nitrogen use while improving efficiency and sustaining productivity.

## Abstract (web summary)

This study evaluates nitrogen surplus reduction scenarios across Europe using century-long sub-national data and a multidimensional clustering algorithm. The findings show that a 20% fertilizer reduction alone is insufficient to meet Farm to Fork targets and that region-specific strategies, combined with advances in technology and management practices, are essential.

## Main

Decades of synthetic fertilizer (hereafter referred to as fertilizer) use in the European Union (EU) have boosted crop productivity but also led to excessive nitrogen (N) pollution, causing algal blooms, biodiversity loss, nitrate contamination and air pollution^1–3^. Agriculture remains a major N pollution contributor, with diffuse sources complicating environmental progress^4^. In response, the EU has implemented several directives^5,6^, and economic and political changes since the late 1980s have curbed intensive fertilizer and manure use on agricultural land^1,7^. Yet many targets remain unmet, and aquatic ecosystems continue to struggle^8^. Recognizing this, the European Commission launched the Green Deal programme^9^, with the Farm to Fork (F2F) Strategy, which focuses on a sustainable transition of the agricultural sector^10^. Among other targets, the F2F Strategy mainly aims to halve nutrient losses by 2030 while maintaining soil fertility and, for this, imposes a reduction in fertilizer use of at least 20%. It also calls for the development of an integrated nutrient management plan to address nutrient pollution from livestock farming.

Previous studies have assessed the feasibility of these targets by estimating N balances and potential N loss reductions under future scenarios^11,12^. While insightful, they often focus on the aggregated EU level and do not fully account for regional variability. However, different regions have differing sensitivities to N pollution due to variations in land use, technology and management practices^8,13^. For instance, fertilizer reductions may be effective in countries like Germany, France and Poland, where it is heavily relied upon for agricultural production^14^, whereas areas with high livestock density, such as Wales and the Netherlands, may benefit more from reducing manure use^15^. Improved N management requires better understanding of these regional profiles. Furthermore, technological and management practices (TMPs), including precision fertilization, smart fertilizers and improved crop varieties, offer promising solutions for enhancing N use efficiency (NUE) and mitigating environmental losses^13,16^, yet their potential in reducing N losses remains underexplored.

This study assesses the effectiveness of agricultural N loss reduction scenarios in achieving the F2F Strategy’s target, considering regional differences and the predominant sources of excess N based on their spatially differentiated historical developments. The F2F nutrient loss target is expressed as a reduction target for agricultural N surplus (Methods)—a key indicator for quantifying N losses and setting a planetary boundary for N flows^17^. Reducing agricultural N surplus is crucial step towards minimizing overall N losses. Here, we go beyond previous studies and examine N surplus reduction scenarios across the EU leveraging century-long (1850–2019) estimation of sub-national N surplus, incorporating uncertainties from underlying data and methods^18^. Using a self-organizing map^19^ (SOM)-informed multidimensional clustering algorithm, we classify Nomenclature of Territorial Units for Statistics (NUTS) 2 regions into distinct typologies based on their historical trajectories in N surplus and land use (Methods). To explore pathways towards achieving the F2F target, we evaluate six plausible N surplus reduction scenarios that span the least to the most ambitious. In addition, we complement this scenario-led (top-down) analysis with a bottom-up investigation to explore the full range of N surplus reduction^20^, thereby recognizing deep uncertainty^21^ in projecting the future changes of N surplus. Importantly, based on our large-range N (input and output) database, we quantify the effects of potential TMP improvements through hyperbolic response functions^13,16^ on plausible N surplus developments. TMP advancements could serve as a key complementary lever, particularly in fertilizer- or manure-dependent regions. Our findings indicate that a 20% fertilizer reduction—a key F2F strategy—alone may be insufficient, highlighting the need for integrated, regionally targeted strategies that combine structural shifts with TMP enhancements.

## Results

### Centennial evolution of N surplus typologies in the EU

**Fig. 1:**
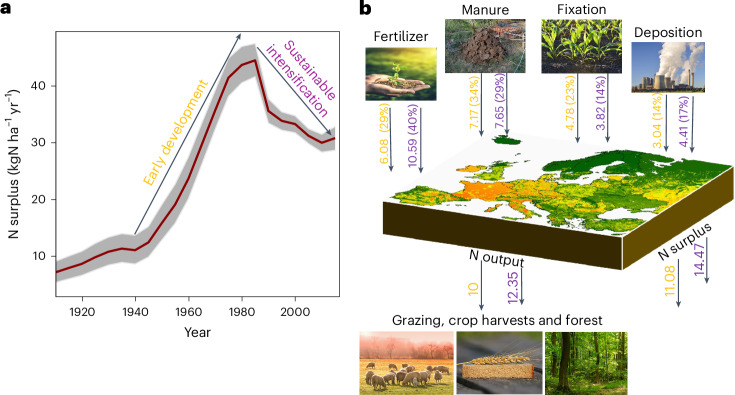
Centennial evolution of N surplus in Europe. **a**, Annual evolution of N surplus (kgN ha^−1^ yr^−1^) at the EU-27 level depicting the early development phase (pre-1980s) and the sustainable intensification phase (post-1980s). The grey ribbon shows the uncertainty range (minimum and maximum values) derived based on the 16 N surplus estimates (Methods), while the average value is represented by the red line. **b**, Mean of 16 N surplus and its underlying components (TgN yr^−1^) at the EU-27 level aggregated during the early development phase (1940–1985, in yellow) and the sustainable intensification phase (1986–2019, in purple). The values (in %) in the brackets show the share of the individual N components in the total N inputs. Basemap data in **b** from ref. ^18^. Credit: images in **b**, Pixabay.com. Source data

The century-long evolution of N surplus at the EU-27 level revealed two phases (Fig. 1a). During the early development phase (approximately 1940 to mid-1980s), N surplus rose sharply, peaking at 45 kgN ha^−1^ around 1985 due to increased fertilizer use to meet food demand. In the subsequent sustainable intensification phase (from mid-1980s to 2019), N surplus declined due to policy interventions (for example, EU Nitrates Directive and Common Agricultural Policy reforms), alongside technological advancements (for example, precision fertilization, improved manure management and adoption of high-efficiency crop varieties^13,22^). Structural shifts, including agricultural intensification in fertile areas and marginal land abandonment, further enhanced NUE^1^. These general patterns are in line with previous findings^22^.

In the early development phase, animal manure accounted for 34% (7.2 TgN yr^−1^) of N inputs, while fertilizers contributed 29% (6.1 TgN yr^−1^; Fig. 1b). After 1985, fertilizers became the dominant source (40%, 10.6 TgN yr^−1^), surpassing manure (29%, 7.7 TgN yr^−1^), driven by their affordability and intensifying crop production^14^. Biological fixation and atmospheric deposition played smaller but consistent roles across both phases. Despite increased N inputs, NUE remained a challenge. While N output (N removal through crops and grass) increased from 10 to 12.4 TgN yr^−1^, its slower growth relative to N inputs reflects persistent inefficiencies in N use. Consequently, NUE slightly declined from 48% to 45%.

**Fig. 2:**
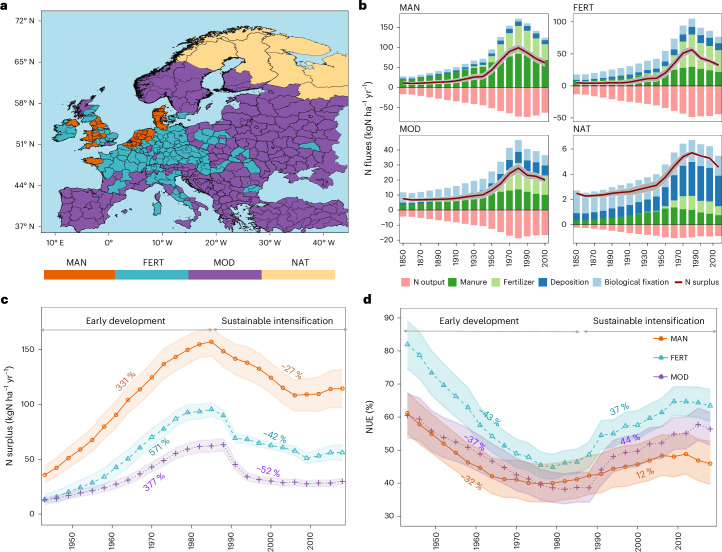
Archetypal N surplus typologies in Europe. **a**, Spatial depiction of the four identified typologies across EU. MAN shows regions dominated by N inputs via animal manure. FERT shows the dominance of synthetic fertilizer inputs. MOD represents moderate inputs from fertilizers and animal manure. NAT indicates the area that includes more natural landscapes compared with agricultural areas and thus is not dominated by agricultural sources. **b**, Decadal trajectory of the total N surplus and its underlying components in different typologies over the period 1850–2019 (all in units of kgN ha^−1^ yr^−1^). The grey ribbon shows the ranges (minimum and maximum values) of the 16 N surplus estimates, while the average value is presented by a red line. The bars indicate decadal mean values for the different N surplus components. The decadal trajectories of 16 individual N surplus estimates along with their underlying components for each typology show a similar temporal pattern as that of the mean N surplus (Supplementary Figs. 9–12). **c**,**d**, Temporal variation of agricultural N surplus (kgN ha^−1^ of agricultural area yr^−1^; **c**) and the corresponding NUE (%; **d**) in the three typologies dominated by agricultural activities (FERT, MAN and MOD). Also indicated are their respective changes (%) in the early development and the sustainable intensification phases. The calculated changes reflect the difference between the endpoint and the starting point relative to the starting point in each of the two phases. The points show the three-year moving average for the period 1920–2019. The ribbons show the ranges (minimum and maximum values) of the 16 N surplus and NUE estimates, respectively, while the average values are shown by darker lines. Source data

Given that synthetic fertilizers now dominate, reducing their use is central to the F2F Strategy for halving nutrient losses by 2030. However, Europe’s diverse agricultural systems create distinct N surplus patterns. Using a SOM clustering algorithm^19^, we identified four N typologies (or clusters) across the EU (Fig. 2a). The animal-manure dominated (MAN) typology predominates in western EU countries like the Netherlands, Denmark, parts of Spain and southern UK entities; the synthetic-fertilizer dominated (FERT) typology is prevalent in central European nations such as Germany and France; the moderate contributions of both manure and synthetic fertilizer (MOD) typology covers east European and Mediterranean countries like Spain, the Balkans, Ukraine and Turkey; and the natural landscapes (NAT) typology is predominant in northern European countries like Norway, Sweden and Finland. The delineation of these typologies also enabled us to assess the effectiveness of a unique N surplus reduction strategy (for example, F2F target of reducing fertilizers) across different European landscapes.

The temporal trends in N surplus follow a consistent pattern across typologies: a rise during the early development phase due to agricultural intensification, followed by a decline in the sustainable intensification phase, influenced by environmental regulations and technological advancements. Both N inputs and outputs increased sharply during the early development phase in all clusters (Fig. 2b), reflecting efforts to meet growing food demand. However, N outputs grew more slowly than inputs, indicating persistent inefficiencies in N use. N surplus levels varied substantially among typologies (Fig. 2b). The MAN cluster had the highest values, increasing from 28 ± 5 in 1940 to 61 ± 7 kgN ha^−1^ yr^−1^ in 2019 (estimates here reflect the mean ± s.d. values based on 16 N surplus time series; see Methods for details). This reflects the dominance of livestock-driven systems in regions with intensive animal farming. The FERT cluster displayed a lower range, from 12 ± 4 in 1940 to 33 ± 3 kgN ha^−1^ yr^−1^ in 2019, aligning with its reliance on synthetic fertilizers. The MOD cluster, with a balanced mix of manure and fertilizer inputs, ranged between 10 ± 2 and 20 ± 1 kgN ha^−1^ yr^−1^ between 1940 and 2019. The NAT cluster consistently had the lowest N surplus, ranging from 3 ± 0.2 in 1940 to 4 ± 0.1 kgN ha^−1^ yr^−1^ in 2019, reflecting its minimal agricultural activity.

Regarding N input sources, the MAN cluster predominantly relied on animal manure, contributing 72% in the 1940s and decreasing to 55% in the 2010s (Fig. 2b). Fertilizer contributed 32% to N input in the 2010s, while biological fixation and atmospheric deposition accounted for 5% and 8%, respectively. Conversely, the FERT cluster saw fertilizer inputs rise from 16% in the 1940s—probably reflecting early sources such as guano and Chilean saltpetre, which were prevalent before the widespread adoption of Haber–Bosch-derived fertilizers^23^—to 45% in the 2010s. During the same period, animal manure contributions declined from 43% to 28%, and biological fixation decreased from 37% to 13%. The MOD cluster had a more balanced distribution, while the NAT cluster saw biological fixation as the main N input until 1950, with atmospheric deposition increasing thereafter. Owing to its dominance by natural vegetation (Supplementary Fig. 1), the NAT typology was excluded from further agricultural N surplus analysis.

Temporal variations in agricultural N surplus and NUE varied across typologies (Fig. 2c,d). During early development (percentage changes were compared between the beginning (1940–1942) and end (1983–1985) intervals), N surplus increased most in FERT (571%), followed by MAN and MOD (330–377%). In the sustainable intensification phase (percentage changes were compared between 1986–1988 and 2017–2019 intervals), MOD had the largest decline (52%), while MAN declined least (27%). NUE fell in all typologies during early development, with FERT dropping (43%). However, NUE improved during the later phase—rising by 12% in MAN, 37% in FERT and 44% in MOD—reflecting improved N management.

These trends result from the interplay of socio-economic policies, land-use patterns, technological advancements, agro-food systems configurations and territorial specialization. Regulations like the EU Nitrates Directive and the EU milk quota probably improved nutrient management by limiting manure application and livestock density, particularly in MAN typology. TMP advancements, including precision fertilization and manure management, improved NUE by optimizing N application and reducing losses^24^. Structural shifts, such as land-use specialization and market-driven intensification, also influences these trends. At a broader scale, country-specific NUE improvements often correlate with economic growth, as higher gross domestic products facilitate greater access to advanced agricultural practices^22^. The historical N inputs and outputs patterns observed across typologies serve as the basis for implementing two TMP scenarios—same TMPs and improved TMPs—to evaluate the role of technology in reducing N surplus (Methods).

### Assessment of N surplus reduction scenarios

Achieving the EU Green Deal’s F2F target of halving nutrient losses by 2030 requires substantial reductions in N surplus. To evaluate the feasibility of this target, we first assess six plausible N surplus reduction scenarios (top-down analysis), which were selected based on their practical applicability. The underlying assumptions and associated reductions in N inputs and outputs for these scenarios are summarized in Table 1 (see Methods for further details). N output changes were estimated under two approaches: (1) same TMPs, reflecting 2015–2019 practices; and (2) improved TMPs, assuming broader adoption of advanced practices. Similar to previous studies^13,16^, a one-parameter (*c*) hyperbolic function was used to capture the yield (that is, N output) response to N input. Improved TMPs simulate shifts in this response due to wider adoption of some existing TMPs (for example, precision fertilization, nitrification inhibitors), or development and adoption of new TMPs (for example, improved cultivars, smart fertilizers)^24^.

**Table 1:** Scenario assumptions and reductions in N input and results for N output in 2030 compared with the baseline (2015–2019)

Scenarios	Assumptions	Reduction in N inputs		Reduction in N outputs	
		Fertilizer (%)	Manure (%)	Same TMPs (%)	Improved TMPs (%)
BAU	^a^	^a^	^a^	^a^	^a^
GD-F	Lower fertilizer under Green Deal	20	^b^	4–7	0–2
FAO-F	Lower fertilizer under FAO	43	^b^	9–15	4–11
FAO-FM	Lower fertilizer and animal manure under FAO	43	4	10–16	6–12
BAF	Lower animal manure through better animal feed	20	10	7–9	3–4
LAP	Lower animal manure by using less animal products	20	20	10–12	6–7

^a^Current trend is extrapolated to 2030 using GAM fitting.

^b^Same as in the baseline (2015–2019).

BAU, business as usual; GD-F, Green Deal Fertilizer, FAO-F, FAO Fertilizer; FAO-FM, FAO Fertilizer and Manure; BAF, Better Animal Feed; LAP, Less Animal Products. N output results show reduction ranges across typologies, using two approaches: the same TMPs and the improved TMPs. Scenarios were selected to estimate N surplus in 2030.

**Fig. 3:**
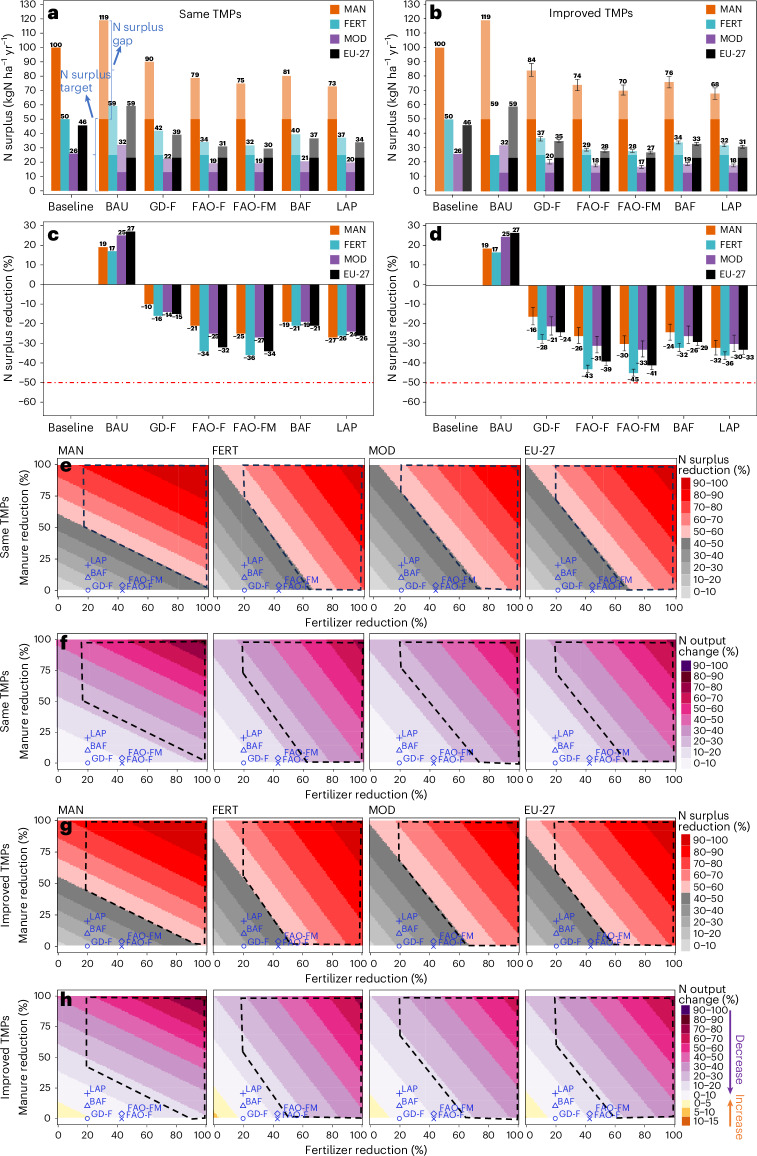
Agricultural N surplus projections for different typologies and EU-27 by 2030 under different intervention scenarios. **a**,**b**, N surplus (kgN ha^−1^ of agricultural area yr^−1^) in baseline, business as usual (BAU) and under specific scenarios (top-down analysis) using the same TMPs (**a**) and the improved TMPs (**b**) approaches. Specific scenarios are: Green Deal Fertilizer (GD-F), FAO Fertilizer (FAO-F), FAO Fertilizer and Manure (FAO-FM), Better Animal Feed (BAF) and Less Animal Product (LAP). The dark shading of the bars shows the target to halve N surplus by 2030, and the light shading of the bars shows the remaining gap to meet the target level. **c**,**d**, N surplus reductions (%) in the BAU and specific scenarios relative to the baseline period estimates with the same TMPs (**c**) and the improved TMPs (**d**) approaches. The red dashed line depicts the N surplus reduction target of 50% according to the Green Deal F2F Strategy. Values on the bars in **b** and **d** are based on the mean estimate of the *c* coefficient for the improved TMPs approach, while error bars reflect the respective 95% confidence interval corresponding to the linear fit of *c* (*n* = eight 5-year estimates). Values on the bars in **a** and **c** represent scenario results under the same TMP approach, using the baseline *c* coefficient from 2015–2019. **e**–**h**, N surplus reductions (%; **e**,**g**) and N output changes (%; **f**,**h**) relative to the baseline estimates (2015–2019) in different typologies and the EU-27 for a whole range of scenarios (bottom-up analysis) considering fertilizer and animal manure reduction under the same TMPs approach (**e**,f) and the improved TMPs approach (**g**,**h**). Each plot represents outcomes for each typology and the EU-27 aggregate (*n* = 4 per scenario). Blue markers highlight the five top-down scenarios shown in **a**–**d**). Dashed polygons represent the desired space where the target of 50% N surplus reduction is achieved while reducing fertilizer by at least 20% according to the Green Deal F2F Strategy. Source data

All scenarios are evaluated relative to the baseline estimates of 2015–2019—this period is in line with the Green Deal recommendation (Methods). N surplus changes under selected scenarios using the same and improved TMPs approach are reported in Fig. 3a–d. In the business as usual (BAU) scenario, contemporary trends of N surplus were projected using a generalized additive model (GAM) fitting (Methods), leading to a 17–27% increase in N surplus across typologies (Fig. 3c,d), confirming that current trends will not meet the F2F target. In the Green Deal Fertilizer (GD-F) scenario—featuring a 20% fertilizer reduction—N surplus declined only 10% (MAN, from 100 to 90 kgN ha^−1^ yr^−1^) to 16% (FERT, from 50 to 42 kgN ha^−1^ yr^−1^) under same TMPs, with 4–6% reductions in N output (Fig. 3c and Supplementary Fig. 2c). With improved TMPs, N surplus reductions increased to 16–28% (95% interval range: 11–30%, reflecting uncertainty in TMP parameterization; see Methods for details), with lower reductions in N output (around 2%), indicating the efficiency gains from technological advancements (Fig. 3b,d). The Food and Agriculture Organization of the United Nations Fertilizer (FAO-F) scenario (43% fertilizer cut) aligns with the Towards Sustainability (TSS) scenario projected for high-income countries (HIC) by FAOSTAT for 2030^25^. It yielded N surplus reductions between 21% (for the MAN typology) and 34% (for the FERT typology) under the same TMPs approach (Fig. 3a,c), and between 26% (22–30%; for the MAN typology) and 43% (41–45%; for the FERT typology) under the improved TMPs approach (Fig. 3b,d). The corresponding N output reductions ranged from 8–15% with the same TMPs and 4–11% with the improved TMPs, depending on the typology (see Fig. 3b,d and Supplementary Fig. 2), showing that improvements in TMPs help mitigate yield losses while enhancing N efficiency. However, this scenario still falls short of halving N surplus in all typologies.

To achieve the halving N surplus target, additional reductions in animal manure would be necessary. We therefore evaluated scenarios combining fertilizer and manure reductions. The FAO Fertilizer and Manure (FAO-FM) scenario considered a 4% decrease in animal manure (following FAO TSS scenario assumptions for HIC^25^) in addition to the 43% reduction in fertilizer from FAO-F. Under the same TMPs, it resulted in N surplus reductions of 25–36% and N output reductions of 10–17%. With improved TMPs, N surplus reductions increased to 30–45%, with N output reductions of 6–12% (Fig. 3a–d and Supplementary Fig. 2). The Better Animal Feed (BAF) scenario includes a 10% manure reduction from feed optimization^26^, in addition to the (20%) reduction in N inputs from fertilizer in GD-F. N surplus reductions ranged from 19–21% (same TMPs) to 24–32% (range: 20–34%; improved TMPs). The Less Animal Products (LAP) scenario reflects reduced livestock numbers via decreased human consumption of animal products in addition to better animal feed, leading to 20% reduction in both manure and fertilizer inputs. It led to 24–27% N surplus reductions under same TMPs, with the greatest in MAN (27%) and lowest in MOD (24%). Corresponding N output reductions ranged from 10% to 12% (Supplementary Fig. 2). With improved TMPs, N surplus reductions increased to 30–36% (range: 25–38%), with the highest gains in FERT and lowest in MOD typologies. Additionally, our analysis of N output from cropland across all scenarios revealed reductions ranging from 5% to 20%, depending on the typology and scenario (Supplementary Fig. 3).

As expected, N surplus reductions were consistently greater under improved TMPs than with the same TMPs approach. Overall, the FAO-FM scenario consistently achieved the highest N surplus reductions under both the same TMPs and the improved TMPs approach, except for MAN, where LAP was more effective (Fig. 3a–d). This highlights the greater potential of TMP improvements over maintaining baseline practices.

**Fig. 4:**
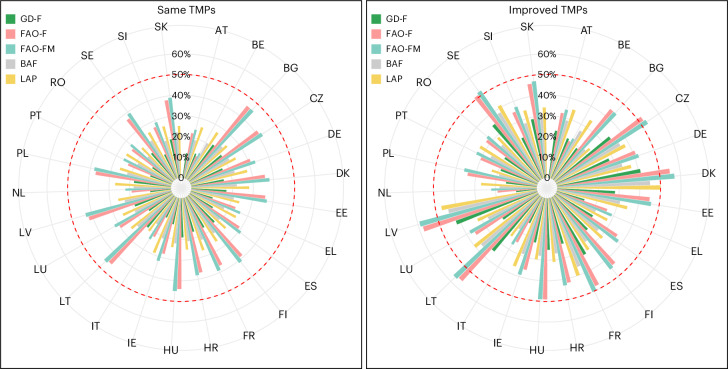
Projected agricultural N surplus reduction by 2030 relative to the baseline estimates (2015–2019) for the EU-27 countries. Shown are the results using the same TMPs and the improved TMPs approaches for five specific scenarios: GD-F, FAO-F, FAO-FM, BAF and LAP. The red dashed circle represents the N surplus reduction target of 50% by 2030 of the EU Green Deal F2F Strategy. Two-letter country codes follow the ISO 3166-1 alpha-2 standard. Complementary plots showing the country-wise estimates of agricultural N surplus (kgN ha^−1^ yr^−1^) for the selected scenarios under the same and the improved TMPs approaches are shown in Supplementary Fig. 4. Source data

**Fig. 5:**
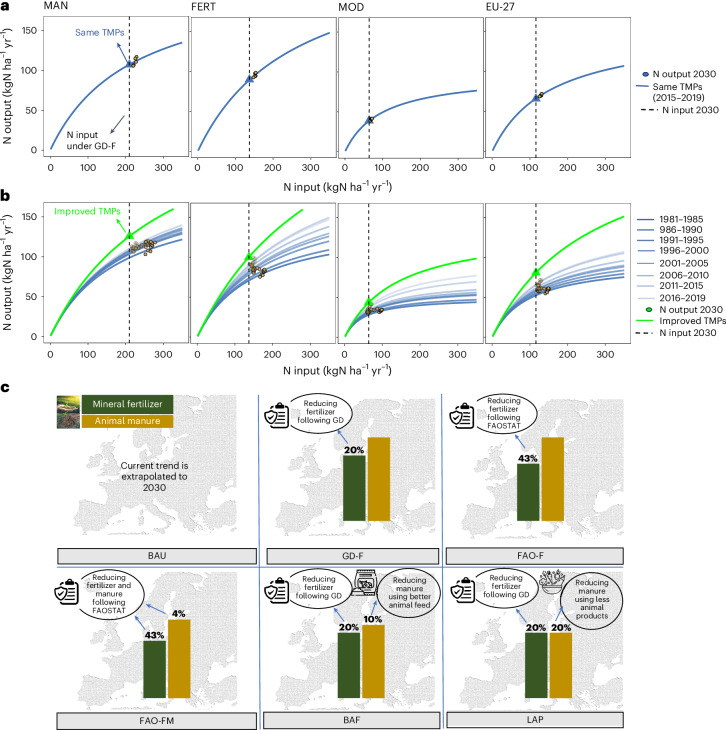
Illustration of the approaches to estimate N output for different typologies under the GD-F scenario and graphical representation of specific N surplus reduction scenarios. **a**, N output in 2030 under the same TMPs approach (that is, using average N input–output relationships from the baseline period 2015–2019). The input–output relationship is represented by a one-parameter hyperbolic function (blue line) fitted to the baseline period (2015–2019), with the blue triangle indicating the projected N output at the reduced N input level assumed under the GD-F scenario. **b**, N output in 2030 for the improved TMPs approach based on the full historical data from 1981–2019. For each typology, multiple one-parameter hyperbolic functions were fitted for eight 5-year periods between 1981 and 2019, shown as shaded blue lines. The improved TMP curve (green line) was derived by linearly extrapolating *c* to 2030 based on its evolution across eight historical intervals (equation (6)). The resulting N output in 2030 under this improved TMPs assumption is shown as green triangles. The dashed vertical line in both panels shows the reduced N input by 2030 under the GD-F scenario, that is, 20% less N input from fertilizer compared with the base year (2015–2019). **c**, Graphical representation of specific N surplus reduction scenarios. The base map shows the European domain and is used for illustrative purposes; it is based on N surplus data from ref. ^18^. Credit: icons in **c**, Canva. Source data

We also assessed country-level N surplus reductions (see Fig. 4 and Supplementary Fig. 4 for the projected range of N surplus). This is critical to inform country-specific nutrient and land management strategies. Under improved TMPs, N surplus reductions ranged from 10% to 59%, while the same TMPs yielded 9–46%. The FAO-F scenario successfully halved the N surplus in Latvia (LV: 58%), Lithuania (LT: 56%), Denmark (DK: 55%) and Sweden (SE: 51%). FAO-FM added France (50%) to this list. Thus, only four to five EU countries would meet the F2F targets under these scenarios (Figs. 4 and 5). As with typologies, reductions vary across countries (Supplementary Figs. 5–8), emphasizing the need for spatially differentiated strategies. Overall, while some countries may achieve the F2F target under the selected scenarios, a 50% N surplus reduction remains unattainable, for most countries and at the typology level.

This limitation highlights the need to move beyond predefined scenarios (top-down analysis). For this, we explore the full range of potential N input reductions to identify which combinations of fertilizer and manure reductions would meet the 50% N surplus reduction target under same and improved TMPs conditions (bottom-up analysis; Fig. 3e–h). We evaluate reductions in N inputs from fertilizers and animal manure application (assuming reduced animal manure production) ranging from 0% to 100% compared with the corresponding baseline estimates of 2015–2019. The target zone (dashed polygons in Fig. 3e–h) reflects the Green Deal’s F2F Strategy of a 20% reduction in fertilizer use and a 50–100% reduction in N surplus.

Under the same TMPs approach, halving N surplus requires substantial fertilizer reductions, namely 100% for MAN, 68% for FERT, 77% for MOD and 72% for EU-27, if manure levels remain unchanged (Fig. 3e). To remain within the target zone, N output reductions would need to reach 23% for MAN and 25% for other typologies and EU-27 (Fig. 3f). Alternatively, if fertilizers remain at a 20% reduction, manure use would need to decrease by 49–79% across typologies. Additionally, Fig. 3a shows that a more aggressive reduction strategy aimed at curbing both fertilizer and animal manure would be required to achieve the target of halving the N surplus. This means that, for example, a 40% reduction in fertilizer combined with corresponding reductions in animal manure (ranging from 38% to 51% across typologies) would be required to achieve the desired target. Under the improved TMPs approach, fertilizer reductions of 96% (MAN), 55% (FERT), 68% (MOD) and 61% (EU-27) are required to halve N surplus while keeping manure levels at the baseline rate (Fig. 3g), which was relatively lower compared with the same TMPs approach. By construct, the minimum N output reduction is lower under the improved TMPs approach compared with the same TMPs approach, with 18% for MAN and EU-27, 16% for FERT and 19% for MOD. These findings also highlight that, for the different typologies, N surplus reductions exhibit different, yet varying levels of sensitivity to changes in N inputs. While this bottom-up analysis identifies the necessary fertilizer and manure reductions to meet the F2F target of halving N surplus, achieving them in practice will require systemic changes beyond TMP improvements. A more integrated approach—where policies simultaneously support improved manure management, promote circular farming practices and encourage dietary shifts—will be critical to make these reductions both feasible and sustainable at the regional level.

## Discussion

Reducing agricultural N surplus is central to the EU Green Deal’s F2F Strategy, which targets a 50% cut in nutrient losses by 2030^10^. Our results show that a 20% fertilizer reduction (GD-F scenario) yields only a 10–16% N surplus cut, consistent with Billen et al.^12^, who found that this scenario would only lower soil N surplus by 20% by 2050. While reducing N surplus is essential for mitigating air, water and soil pollution^1,17^, achieving this target requires systemic transformations in the entire agro-food system^11,12^. Furthermore, the EU’s reliance on fertilizer imports—particularly from Russia and Belarus—highlights the urgency of reducing external dependencies^14^. Transitioning towards resilient, regionally self-sufficient farming systems through circular agricultural practices could mitigate these dependencies while ensuring long-term sustainability^12^. However, such shifts demand significant investments, policy support and stakeholder collaboration. Additionally, reducing N inputs could lower domestic crop yields, increasing reliance on food imports to compensate for losses and shifting environmental burdens to countries with weaker regulations and less sustainable agricultural practices^27^, counteracting the intended benefits of the F2F Strategy. Avoiding this demands an integrated approach combining trade policies, dietary shifts and food waste reduction to minimize pollution outsourcing while maintaining agricultural productivity^11,12,27^. Here we define such integration as alignment of N input reductions with improved TMPs and broader system-level measures, including trade, consumption and waste management interventions^1,28^.

Europe’s diverse agricultural landscapes discourage uniform implementation of the F2F Strategy. Recognizing this, we classified EU regions into four typologies—MAN, FERT, MOD and NAT—based on the historical trajectories in N surplus sources and land-use developments. Each typology reflects the dominant N surplus source. This classification aligns with Billen et al.^12^, who also identified livestock-dense regions like the Netherlands and Denmark in their EU-wide agro-food transitions. While their analysis presented scenarios at an aggregated EU level, our study takes a spatially differentiated approach, capturing long-term regional variations in N surplus. Even within these broad typologies, substantial regional differences exist, as indicated by Rodríguez et al.^28^, who clustered Spanish NUTS-3 regions into distinct agro-food system categories. This highlights the need for further finer-scale, targeted mitigation strategies within typologies. In MAN typology, advanced manure management (for example, anaerobic digestion, precision spreading) is essential to handling excess manure^11^. FERT regions, prevalent in central Europe, could benefit from precision agriculture and legume-based rotations to reduce synthetic fertilizer dependence^29^. MOD systems require a combination of manure management and diversified cropping to optimize nutrient use. Beyond typology-specific strategies, reconnecting crop and livestock systems at regional scales can help reduce N surplus^30^. This approach improves manure use, reduces reliance on fertilizers and imported feed, and enhances NUE. Given that our typologies reflect long-term specialization, such territorial strategies can complement technical measures and support more balanced nutrient flows.

Our bottom-up analysis reveals that halving N surplus requires substantial reductions in fertilizer and manure inputs, with some typologies needing up to 100% fertilizer reduction if manure remains unchanged. Although essential for meeting the F2F target, such reductions face numerous barriers. Reducing fertilizer use offers multiple benefits, including cost savings for farmers^31^, improved economic and environmental sustainability^31^ and reduced exposure to harmful chemicals^32^. However, abrupt reductions threaten yields and economic stability in fertilizer-dependent regions^33^. Geopolitical factors, such as rising fertilizer costs driven by the COVID-19 pandemic and the Russian invasion of Ukraine^34^, have further disrupted food systems, highlighting the risks of import dependence. Industry resistance further complicates efforts to phase-out fertilizers^34,35^. Beyond fertilizer reductions, dietary shifts offer another pathway for reducing N surplus. The LAP and FAO-FM scenarios indicate that reducing livestock production through dietary shifts can decrease feed demand, free up land for alternative food production and reduce Europe’s reliance on imported feedstocks^12^. However, these shifts face economic resistance from livestock-dependent economies (for example, Germany, Ireland and Denmark)^36^ and societal resistance due to consumer preferences, accessibility issues, the perceived nutritional adequacy of meat-free diets and the limited knowledge about plant-based alternatives^37^. The BAF scenario, which optimizes animal feed to reduce N losses, offers environmental benefits^38^ but reducing protein content in feed beyond a certain threshold may compromise animal health and productivity^39^. Additionally, alternative feed formulations are often more expensive than conventional options, creating financial barriers for farmers^40^.

TMPs are critical for improving NUE while minimizing trade-offs between N surplus reductions and maximizing crop yields^22^. Our assessments indicate crop yield reductions of 5–20%, depending on the typology and scenario, with smaller reductions under improved TMPs (1–15%), aligning with Billen et al.^12^, who reported crop yield reductions of approximately 6–20% in their investigated scenarios. Historical trends indicate slow gains in maximum achievable N outputs (*c* values)^13,16^, making it challenging to accelerate progress beyond the rates achieved in our improved TMPs approach. This implies that, even with advanced TMP adoption, some yield loss would be unavoidable to meet the F2F target. Importantly, geophysical constraints—such as soil quality and climate variability—may limit TMP effectiveness in certain regions, requiring region-specific adaptations^41^.

Overall, our study provides a comprehensive assessment of N surplus reduction scenarios within the EU Green Deal and F2F Strategy framework. Halving N surplus remains challenging due to the need for rapid changes in agricultural practices, financial constraints and short time frames. Achieving the F2F goals of reducing N losses would require region-specific efforts to reduce N inputs in agricultural areas, while simultaneously increasing NUE through improved TMPs to maintain agricultural productivity. These findings emphasize that even technically achievable N surplus reductions may not be practically feasible without broader system-level support—such as aligning agri-environmental policies with N efficiency goals, promoting sustainable trade standards, and enabling dietary shifts and food waste reduction—particularly in livestock- and fertilizer-intensive regions^11,42^. Policies and shifts in (social) dietary habits may therefore need to be adapted to incentivize farmers and facilitate the adoption of practices to reduce N surplus^43^. Moreover, N surplus reduction intersects with other sustainability goals, including lower greenhouse gas emissions and improving soil health, requiring integrated policy coordination^9^. Although our analysis uses N surplus as a proxy for potential N losses, future research could more explicitly consider the underlying biophysical processes affecting N retention and loss—such as changes in soil organic matter or microbial activity—especially under large input changes^44^, as well as N loss pathways like leaching, volatilization and denitrification under different management practices and input regimes. Furthermore, a more comprehensive approach is required to translate the N surplus reductions into resulting N (pollution) benefits in different terrestrial compartments including water, soil and air^17^. Overall, achieving N losses reduction under the EU Green Deal will require coordinated efforts addressing the complex social, economic and environmental factors of N management across Europe’s diverse landscapes.

## Methods

### N surplus

In this study, we used the century-long, sub-national dataset of individual components of N (surplus) budget and land use to classify Europe into different typologies at NUTS 2 level, as those are basic regions for regional policies^45^. The underlying methodology used to construct this dataset is detailed in ref. ^18^. Essentially, the N surplus dataset provides 16 N surplus estimates for both agricultural and non-agricultural soils across Europe at a 5-arcmin spatial resolution for more than a century (1850–2019). Following the soil–plant system boundary framework^46^, we define N surplus (Surp) as the difference between N inputs (In) and N outputs (Out), as given in equation (1). The soil surface budget approach is used, which excludes gaseous losses occurring during manure storage, ensuring that manure inputs represent only the amount applied to soils^47^. N inputs consist of fertilizers (In_Fert_), manure (In_Man_), atmospheric deposition (In_Dep_) and biological fixation (In_BNF_), as given in equation (2). N outputs refer to N removal via harvested crops (Out_crops_) and via animal grazing and cutting of grass (Out_past_; all variables are in kgN ha^−1^ yr^−1^).

1 $${\rm{Surp}}(i,y)={\rm{In}}(i,y)-{\rm{Out}}(i,y)$$

2 $${\rm{In}}(i,y)={\rm{I{n}}}_{{{\rm{Fert}}}}(i,y)+{\rm{I{n}}}_{{{\rm{Man}}}}(i,y)+{\rm{I{n}}}_{{{\rm{Dep}}}}(i,y)+{\rm{I{n}}}_{{{\rm{BNF}}}}(i,y)$$

3 $${\rm{Out}}(i,y)={\rm{Ou{t}}}_{{{\rm{crops}}}}(i,y)+{\rm{Ou{t}}}_{{{\rm{past}}}}(i,y)$$

where *i* is grid cell and *y* is year.

The total N surplus includes contributions from both agricultural and non-agricultural areas. We utilized total N surplus to classify EU landscapes into four typologies, providing insights into both agriculture- and non-agriculture-dominated regions across Europe. Our detailed analysis focused on agriculture-dominated typologies, taking into account the corresponding N surplus as it is the key driver of environmental impacts and is heavily influenced by inputs such as fertilizers and manure. Agricultural N surplus is calculated as the N inputs to cropland and pastures minus the N outputs removed through crop and pasture harvests. Non-agricultural N surplus accounts for N inputs from fixation and atmospheric deposition to non-agricultural areas (for example, forests, semi-natural vegetation and urban landscapes) minus the N outputs from forest harvests. Please refer to ref. ^18^ for more details on the underlying methodology to construct (agriculture and non-agriculture) N surplus and its components. Further descriptions of different components of N surplus utilized here are provided in the Supplementary Information.

Overall, we accounted for uncertainties due to different underlying data and methodological choices in components of N surplus that are reported to have considerable uncertainties^48^, namely N inputs from fertilizer and manure, and N removal from pastures. Specifically, we used 16 time series of N surplus estimates by combining two estimates for fertilizer, four estimates for animal manure and two estimates for the N removal from pastures, to consider the inherent uncertainty in their reconstructions. Furthermore, we include these N surplus budget components in the typological classification as part of a multivariate SOM classification.

### N surplus typologies

In this study, we used a multidimensional clustering approach to classify the EU landscapes into distinct typologies of N surplus based on the different components of N surplus and land-use trajectories. The classification algorithm is SOMs, also called a Kohonen network. SOMs are a type of artificial neural network that is trained through unsupervised learning to map high-dimensional data onto a two-dimensional grid^19^. Inspired by the topological maps of the sensory processing areas of the brain, where neurons responding to similar inputs are spatially very close to each other, SOMs train randomly assigned weight vectors to map similar input data points to nearby neurons. This results in a visualization of the input data where similar data points are grouped together. SOMs are useful for dimensionality reduction, visualization and clustering, and have been used in a variety of domains. In this study, we applied a single-layer SOM method consisting of a SOM with 2 × 2 = 4 nodes. Twelve variables at NUTS 2 level were used as input data for the classification: N input from fertilizers (two datasets), animal manure (four datasets), fixation, deposition, N output (two datasets) and agricultural and non-agricultural area.

For each of the 393 NUTS 2 regions, we calculated the decadal mean of each variable from 1850 to 2019, focusing on key trends and developments in N surplus (and NUE) during the past 100 years (1920–2020). This centennial period captures the onset of fertilizer use and other agricultural changes that further shaped N surplus evolution, leading to intensification in the mid-twentieth century. We thus divided the domain into four typologies labelled MAN, FERT, MOD and NAT. The optimal number of typologies is determined by the Davies–Bouldin index, which indicates that the cluster is well separated at a minimum value^49^. Previous studies have found that there is no unified method for determining optimal clusters^50^. To address this, we complemented the Davies–Bouldin index analysis with visual inspection of the resulting four typologies. We confirmed that increasing the number of typologies beyond four did not yield distinct N surplus typologies.

### N surplus reduction scenarios

To evaluate pathways for halving N surplus by 2030, we applied two complementary approaches: a top-down and a bottom-up analysis.

We first conducted a top-down analysis, focusing on six specific N surplus reduction scenarios selected for their policy relevance and practical feasibility. These include a BAU scenario—reflecting current trends in N surplus—along with five targeted reduction scenarios. Two scenarios involve reductions in fertilizer use only (GD-F and FAO-F), while the other three include combined reductions in both fertilizer and manure inputs (FAO-FM, BAF and LAP). The rationale and assumptions underlying these scenarios are detailed in ‘Top-down scenario story lines’ (see also Fig. 5c for a visual summary).

After evaluating these scenarios, we found that while a few individual countries may reach the 50% N surplus reduction target, none of the four typologies achieve the intended reduction. This limitation led us to conduct a bottom-up analysis^20,21^ to explore the full range of potential fertilizer and manure input reductions. This approach accounts for the deep uncertainty in future system developments^21^, by covering a wide range of input reductions corresponding implicitly to a variety of socio-economic conditions and management practices. It allowed us to identify demarcate the N input combinations that could achieve the 50% N surplus reduction target.

These two approaches compliment each other in a manner that the top-down analysis evaluates a set of policy-relevant scenarios, while the bottom-up analysis maps the full solution space of N input reductions. By placing the selected scenarios within this broader space, we can understand what additional N input reductions may be needed to meet the intended N surplus reduction target (for example, 50% compared with baseline value) along with their likely responses to N outputs (for example, crop production).

Both the top-down and bottom-up approaches rely on the same method for estimating N output from N input levels, under different TMP approaches (see ‘Estimation of N output under TMP assumptions’).

### Top-down scenario story lines

The BAU scenario assumes that the N surplus in 2030 follows the past trend during 1961–2019 without additional measures. To project N surplus in 2030, we used a GAM fitted to an N surplus dataset covering the period from 1961 to 2019 for all typologies. Unlike traditional linear regression models, GAMs allow for the use of smoothing functions to capture nonlinear behaviours^51^. GAMs have been used to model long-term trends in time series^52^ and to evaluate water quality data over a 32-year period to assess the effectiveness of nutrient reductions^53^. In the GAM model, we used the negative binomial family and log link function, which are appropriate for data with over-dispersion, such as our N surplus data (see Supplementary Fig. 13 for the depiction of GAM model fitting to N surplus data for each cluster and EU-27). We used a thin-plate regression spline as the smooth function to capture the nonlinear trends in the relationship between year and N surplus. By using a spline function, we avoided the assumption of linearity, which is often unrealistic in real-world data.

The GD-F scenario assumes a 20% reduction in fertilizer use by 2030, in line with the F2F Strategy’s target of the EU Green Deal^10^. Note that for each of the N input reduction scenarios we employed both TMP approaches for the corresponding changes in N outputs.

The FAO-F scenario explores the potential reduction in N surplus by 2030 through a reduction in fertilizer use. This scenario is motivated based on FAOSTAT’s TSS scenario for HIC^25^. This scenario reflects a future with better social, environmental and economic factors, leading to a more equitable society with a sustainable agricultural system. The TSS scenario encompasses various assumptions, including a 43% reduction in fertilizer use compared with the 2012 baseline, along with a 5% increase in agricultural production in HIC. Drawing on the TSS scenario, our analysis focuses on assessing the impact of reducing 43% of fertilizers on N surplus. The corresponding changes in N outputs are derived based on both TMP approaches.

The FAO-FM scenario extends the reduction of N inputs beyond the reduction in fertilizer use under the FAO-F scenario by incorporating the reduction in animal manure input by 4%. The reduction value for animal manure (that is, 4%) was derived from the values reported for the reduction of animal products based on the FAOSTAT’s TSS scenario for HIC^25^.

The BAF scenario includes a reduction in N input from fertilizer similar to the GD-F scenario, along with a further reduction in N input from animal manure by 10% due to the reduction in animal manure production resulting from improved animal feeding. This scenario is based on the assumption that, by providing animals with feed that has a lower protein content, farmers can reduce N excretion without compromising animal productivity^26,54^. The reduction in N inputs from animal manure is supported by a previous study^54^, which indicated that a 10% reduction in protein content in animal feed led to a corresponding 10% decrease in N excretion, while maintaining animal productivity. Similarly, other studies found that enriching animal feed with amino acids and reducing its protein content by 1% could lead to a 8–10% reduction in N excretion. In addition, it was found that a reduction in the protein content of the feed of dairy cows resulted in a 14% reduction in N excretion^55^.

The LAP scenario assumes a 20% reduction in N inputs from animal manure, which is driven by both improved animal feeding efficiency and a shift towards plant-based diets. The first 10% reduction stems from optimizing animal feed composition, as included in the BAF scenario, where reducing protein content in feed lowers N excretion without compromising animal productivity. The second 10% reduction assumes a change in human diet towards a more plant-based diet, resulting in less consumption of animal meat and thus translating to less livestock production and corresponding manure. It builds on the findings of previous studies^7,38,42^ that suggest that reducing consumption of animal products and incorporating more plant-based sources not only benefits the environment but is also healthier. For instance, the UK Climate Change Committee recommended a 20% reduction in meat consumption by 2030 to meet net zero emission targets, rising to 35% by 2050, highlighting both environmental and dietary motivations^56^. In addition, the LAP scenario is consistent with that proposed by Bodirsky et al.^26^, which recommends limiting the consumption of animal products to no more than 15% of calories and 29% of protein in any country. Similarly, Leip et al.^11^ also suggested reducing N surplus at European level by 2030 through switching to a vegetarian diet, which would eventually lead to a decrease in N inputs from animal manure. This kind of dietary shift is also presented in the ‘dietary shift scenario’ analysis by Liu et al.^57^, which aims to reduce the consumption of animal products by switching to a balanced diet in 2030 for reduced N losses. More recently, Billen et al.^12^ also proposed a structural transformation of the agro-food system through an ‘agro-ecological scenario’, which assumes a human diet with reduced animal protein intake (30% of total protein consumption compared with the current 58%).

### Estimation of N output under TMP assumptions

We used a one-parameter hyperbolic function—a typical form of yield response function widely used in literature^13,16^ to characterize the relationship between N output and N input (equation (4)):

4 $${\rm{Out}}(i,t)=\frac{c(i,t)\times {\rm{In}}(i,t)}{c(i,t)+{\rm{In}}(i,t)}$$

where *i* represents different typologies, *t* is the time period, *c* is the coefficient of the one-parameter hyperbolic function parameterizing the TMP levels, Out is N output and In is N input, averaged over a given time period (for example, 2015–2019). This function captures the relationship between N inputs and N outputs and allows us to estimate the maximum achievable N output for a given TMP^13,16^, represented by the coefficient *c* (all variables are in kgN ha^−1^ yr^−1^).

In this study, we tested two scenarios of yield (that is, N output) response to N input. The first scenario (same TMPs) assumes that TMPs in agricultural production stay at the level of the most recent period, therefore the yield response to N input does not change from the baseline period (2015–2019). In this scenario, the coefficient *c*(*i*, *t*) in equation (4) is equal to the *c* derived from the record in the period 2015–2019 (equation (5)):

5 $$c(i,t)={c}_{{{\rm{baseline}}}}(i)$$

We then used the N output response functions as in equation (4) with the derived *c* coefficient to estimate N output in 2030, based on the adjusted N input levels defined by the scenario configurations (Fig. 5a).

The second scenario (improved TMPs) assumes that TMPs continue to improve following the pace observed in the past decades. Therefore, the yield response function will be different from the historical period. To parameterize the yield response for this scenario, we first estimate coefficient *c*(*i*, *t*) from 1981 to 2019 using N inputs and outputs over each of the seven 5-year intervals and the last 4-year interval (1981–1985, 1986–1990,…, 2016–2019). The parameter *c*(*i*, *t*) is therefore adjusted dynamically:

6 $$c(i,t)={c}_{{{\rm{baseline}}}}(i)\times (1+\Delta {c}_{{{\rm{TMP}}}}(t))$$

where *c*_baseline_ is the historical value of *c*(*i*, *t*) from the baseline period (2015–2019) and Δ*c*_TMP_(*t*) represents the advancements in TMPs over time.

The time period in the improved TMPs was selected to cover the entire span of the sustainable intensification phase. Then, following the assumption of TMPs’ continues improvement, we extrapolate the *c* values from the historical period to 2030 (see Fig. 5b for more information). This represents one of the possible futures of the yield response to N input under continuous improvement of technologies and increasing adoptions of technologies and practices. The TMPs include, but are not limited to, precision fertilization, nitrification inhibitors and improved crop varieties^13,24^. The coefficient *c* represents an aggregate measure of these advancements, and its extrapolation provides a moderate, evidence-based projection of improved TMPs. However, future advancements may vary depending on socio-economic and policy developments. Using the extrapolated value of *c*, we estimated N output corresponding to the projected N input values in 2030 for each typology using equation (4). The values for the coefficient *c* (kgN ha^−1^ yr^−1^) for both the same and the improved TMPs approach are shown in Supplementary Table 1. While the improved TMPs approach reflects a continuation of sustainable intensification, we recognize the potential for greater improvements through technology transfer or policy-driven interventions^58^. Although exploring all possible technology improvement is beyond the scope of this study, the methods established herein can be tested and further elaborated when corresponding yield response function parameters become available.

To account for the uncertainty around the extrapolation of TMP function (*c* parameter) in the future (2030), we derived and included the upper and lower bounds, reflecting 95% confidence intervals, from this model for each typology. These bounds were used to generate a plausible range of N output responses and the resulting N surplus changes under the improved TMPs scenario for a whole range of combinations of N inputs. Among typologies, MAN showed the highest uncertainty in projected N surplus changes, suggesting that livestock-intensive systems are more sensitive to TMP parameterization than other typologies (Supplementary Table 1). The uncertainty ranges are further visualized in Supplementary Figs. 14 and 15, which show N output and N surplus estimates across all scenarios. Uncertainty analyses are also shown for the selected scenario-based figures (for example, Supplementary Figs. 2 and 3, and Fig. 3), illustrating how results vary under different assumptions of TMP effectiveness. Additionally, Supplementary Fig. 16 provides a complementary analysis showing the difference in N output between same and improved TMPs scenarios across all input combinations, further illustrating the yield benefits from TMP adoption under uncertainty. At the country level, Supplementary Figs. 17– 21 provide further detail on the variability in N surplus projections.

### Reporting summary

Further information on research design is available in the Nature Portfolio Reporting Summary linked to this article.

## Supplementary information

Supplementary Information Supplementary Methods, Supplementary Figs. 1–21 and Supplementary Table 1.

Reporting Summary

Supplementary Data Source data for Table 1. Scenario-specific assumptions and percentage reductions in N inputs (fertilizer and manure) and projected N output ranges under same and improved TMPs, relative to 2015–2019.

## Source data

Source Data Fig. 1 Combined data file for all panels in Fig. 1. Includes N surplus estimates, cumulative input/output components and full raw data for the EU-27 over time.

Source Data Fig. 2 Combined data file for all panels in Fig. 2. Contains cluster assignments and decadal trends of N surplus and NUE across typologies.

Source Data Fig. 3 Combined data file for all panels in Fig. 3. Provides scenario-based projections of N surplus and inputs/outputs by typology for 2030.

Source Data Fig. 4 Combined data file for all panels in Fig. 4. Contains country-level and scenario-specific N surplus values and reduction percentages across the EU.

Source Data Fig. 5 Combined data file for all panels in Fig. 5. Includes baseline and projected N input/output estimates by typology and scenario assumptions.

## Data Availability

The datasets used in this study are publicly available in archived Zenodo repositories. The primary dataset (version 1.0) used to generate Figs. 1–5, along with supporting materials, is available via Zenodo at 10.5281/zenodo.15520404 (ref. ^59^). This includes: (1) all input datasets in .xlsx format; (2) output plots in publication-quality .pdf format; and (3) README files describing variables, units and structure. The underlying nitrogen surplus dataset is available separately via Zenodo at https://zenodo.org/records/6581441 (ref. ^60^). Source data are provided with this paper.

## Code availability

The code used for data processing, analysis and figure generation is available via Zenodo at 10.5281/zenodo.15520404 (ref. ^59^) and is also available via GitHub at https://github.com/batool-masooma/N_surplus_typologies_Europe. Instructions for reproducing the results are provided in the accompanying README files.
