# Supplementary material for: Scenario analysis of nitrogen surplus typologies in Europe shows that a 20% fertilizer reduction may fall short of 2030 EU Green Deal goals
Source: Nat Food. 2025 Aug 19;6(8):787–98. doi: 10.1038/s43016-025-01210-2 (PMC12367536; doi:10.1038/s43016-025-01210-2)
Supplement: Supplementary file 1 — Supplementary Methods, Supplementary Figs. 1–21 and Supplementary Table 1. [file 43016_2025_1210_MOESM1_ESM.pdf]

nature food

Supplementary information https://doi.org/10.1038/s43016-025-01210-2

Scenario analysis of nitrogen surplus typologies in Europe shows that a 20% fertilizer reduction may fall short of 2030 EU Green Deal goals

In the format provided by the authors and unedited

Contents

S1 Supplementary Methods **2**

Underlying components of Nitrogen surplus 2

Fertilizer 2

Animal manure 2

Atmospheric deposition and biological fixation 4

N removal 5

S2 Supplementary Figures **5**

S3 Supplementary Table **5**

S1 Supplementary Methods

Underlying components of Nitrogen surplus

Fertilizer: Fertilizer application data from 1961 to 2019 was obtained from FAOSTAT[1]. For the period 1925–1960, global fertilizer production dynamics provided by Holland et al. (2005)[2] were applied to available country-level estimates for 1961 to estimate historical fertilizer application amount. These dynamics likely encompass early sources of N inputs, including guano, Chilean saltpeter (sodium nitrate), mined N compounds, and early synthetic fertilizers, which were prevalent before the widespread adoption of Haber-Bosch derived fertilizers[3]. Country-level application rates for crops and grasslands were derived from IFA[4] and combined with crop and grassland areas from FAOSTAT[5] to estimate respective application rates. To account for spatial variability, country-level application rates of crops and grassland were multiplied by their gridded areas[6]. To partition fertilizer application amounts to croplands and pastures, two different sets of application rates were considered. In the first approach, the fertilizer application rates based on IFA were adjusted to ensure consistency with country-level fertilizer application from FAOSTAT[1]. In the second approach, these rates were further adjusted to ensure consistency with the partitioning information provided by Einarsson et al. (2021)[7]. Total gridded fertilizer amount applied to soil was obtained by summing fertilizer applied to croplands and pastures for each of the two approaches.

Animal manure: For N inputs from animal manure, we utilized two datasets: FAOSTAT[8] and Einarsson et al. (2021)[7], which provide country-level estimates of manure applied to soil and left on pasture during 1961–2019. These datasets differ in their methodologies for estimating N excretion from livestock. FAOSTAT calculates total N excretion by multiplying country-level livestock counts with regional-level values of typical animal mass and N excretion rates from the Intergovernmental Panel on Climate Change (IPCC)[9]. In contrast, Einarsson et al. (2021)[7] employs an approach based on Lassaletta et al. (2014)[10], which assumes that N excretion rates within each livestock category are proportional to slaughter weights. These rates are then multiplied by livestock counts to calculate total N excretion. Both datasets were incorporated into our analysis, focusing exclusively on manure applied to soils while excluding volatilization losses during manure management and storage. These volatilized forms of N were separately accounted for as part of atmospheric N deposition in the overall N budget.

To downscale country-level manure data to a gridded level, we utilized manure production estimates from Zhang et al. (2017)[11], which were based on the spatial distribution of livestock counts in the Global Livestock Impact Mapping System (GLIMS)[12] and IPCC-based N excretion coefficients. Zhang et al. (2017)[11] provides gridded manure production data at a 5-arcminute resolution for the period 1860–2014. For years before 1860 and after 2014, manure production was assumed constant, equal to the earliest (1860) and latest (2014) available estimates, respectively. Ratios of manure applied to soil and left on pastures to total manure production were calculated at the country level for each dataset

by combining the gridded manure production of Zhang et al. (2017)[11] with grid cell area. These country-level ratios were then applied to the gridded manure production data to estimate gridded rates of manure applied to soil and left on pastures for 1961–2019. For the period 1850–1960, the ratios from 1961 were applied to estimate gridded manure application and pasture rates.

Further, to distribute manure applied to soil between croplands and pastures, we employed two methodologies to address uncertainties. The first method, following Tian et al. (2018)[13], assumed uniform manure application rates within each grid cell, where manure applied to cropland and pasture was proportional to the respective land areas. The second method used country-level proportions of manure application to cropland and pasture derived from Einarsson et al. (2021)[7], based on national statistics and expert estimates for different livestock categories. These proportions were used to adjust the grid-level distribution of manure between cropland and pasture. Overall, four different estimates of gridded manure were derived by utilizing two different input data sources of manure and two different methodologies to distribute manure applied to soil between croplands and pastures.

Atmospheric deposition and biological fixation: For N deposition, the National Center for Atmospheric Research (NCAR), Chemistry-Climate Model Initiative (CCMI) N-deposition monthly dataset[14] was downscaled to a resolution of 5 arcmin and aggregated annually. To estimate N deposition in cropland and pasture, the total N deposition was

multiplied by the proportion of land area per grid cell. N fixation over cropland and pasture was derived by multiplying the respective areas with their N fixation rates.

N removal: For cropland, the N removal was calculated by multiplying the crop production with a crop-specific N content, as derived from existing studies and specified in Batool et al. (2022)[15]. For grasslands/pasture, N removal was calculated assuming a fixed NUE.

S2 Supplementary Figures

S3 Supplementary Table

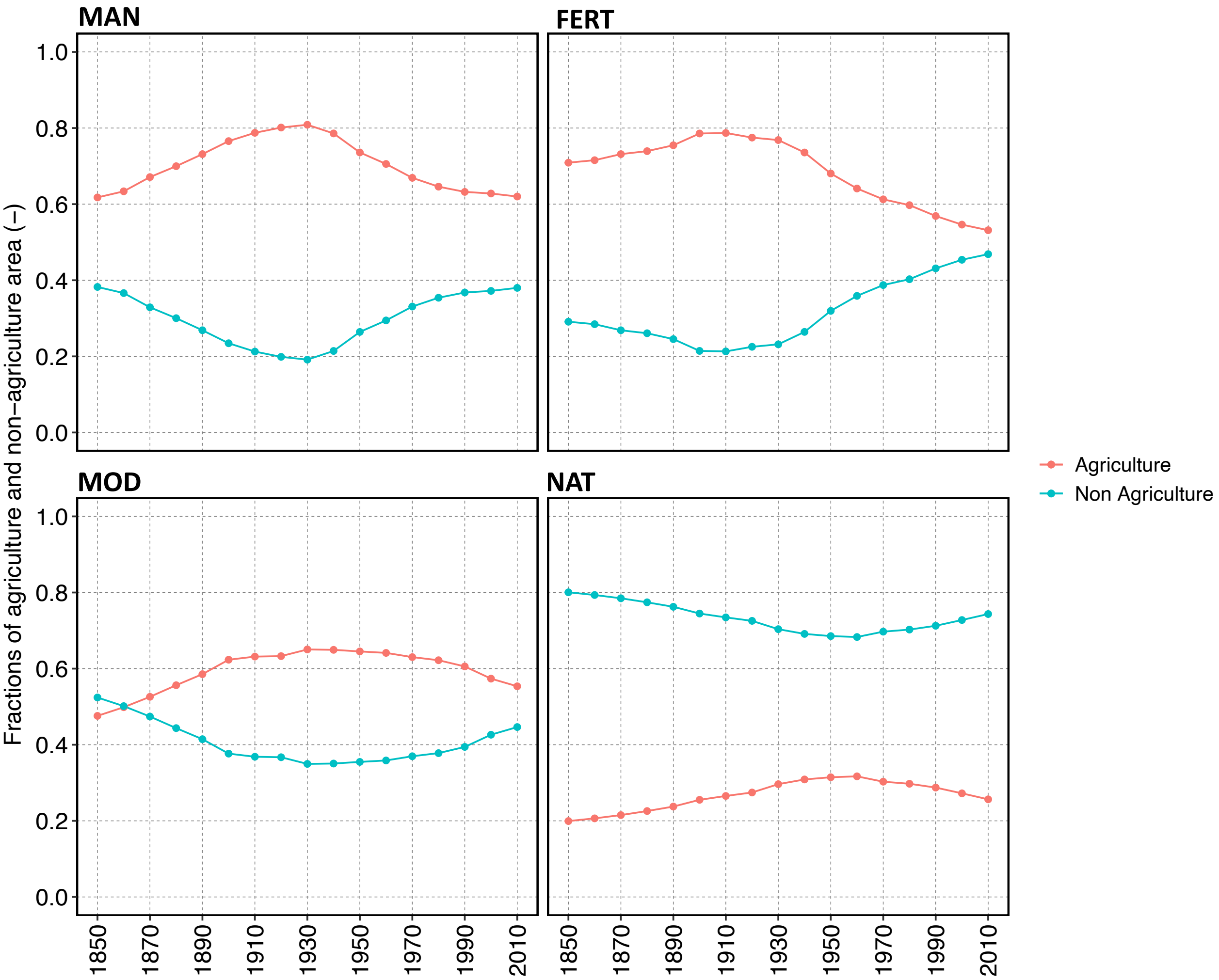

Figure S1: **Long-term time series of of agricultural and non-agricultural land from 1850 to 2019 in four typologies: MAN, FERT, MOD, NAT**. This figure illustrates the decadal averages, with the orange lines/dots representing the proportion of agricultural land and the blue lines/dots representing non-agricultural land. Agricultural land dominates in all typologies with the exception of the NAT typology.

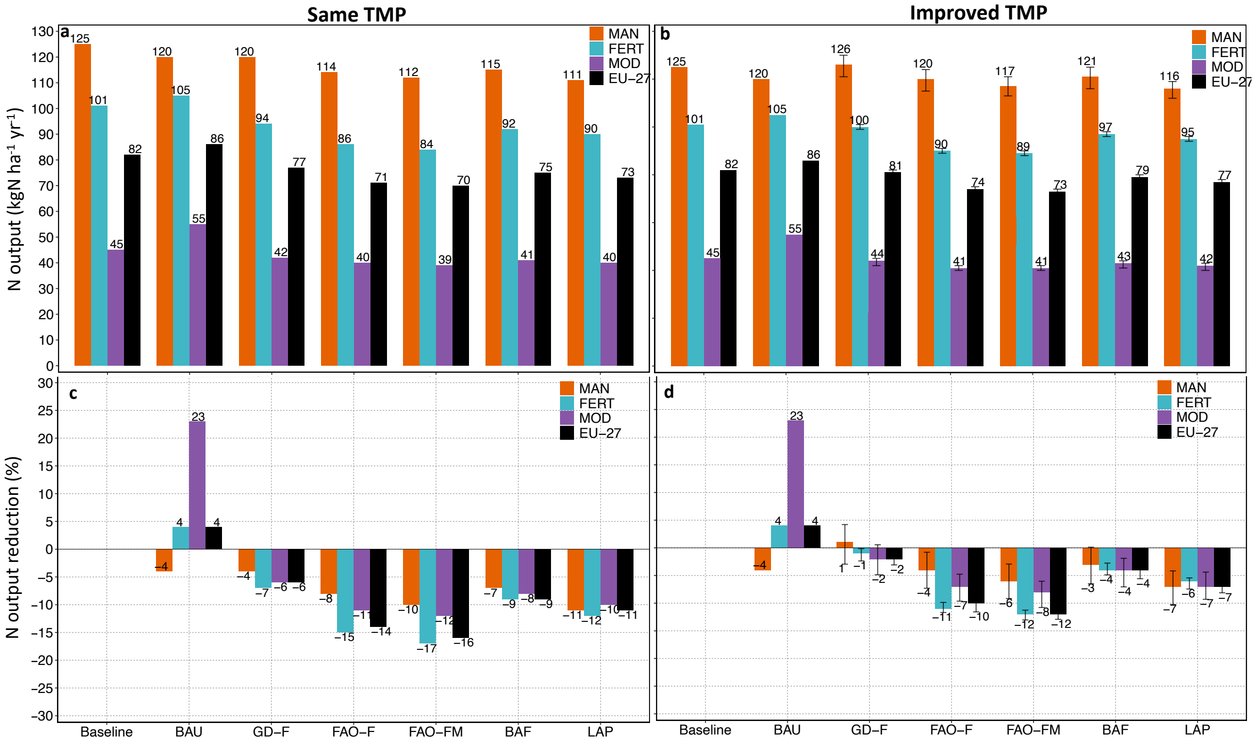

Figure S2: **Agricultural N output projections for different typologies and EU-27 by 2030 under different intervention scenarios**. **(a,b)** Agricultural N output (kgN ha^{-1} of agricultural area yr^{-1}) in baseline, business-as-usual (BAU), and under specific scenarios using the same TMP and the improved TMP approaches, respectively. Results corresponding to the specific five N surplus reduction scenarios are represented by points in the panels (a,b) as: GD-F (Green Deal Fertilizer), FAO-F (FAO Fertilizer), FAO-FM (FAO Fertilizer and Manure), BAF (Better Animal Feed), and LAP (Less Animal Product). **(c,d)** Agricultural N output changes (%) in the BAU and specific scenarios relative to the baseline period estimates with the same TMP and the improved TMP approach, respectively. Values on the bars in panels (b) and (d) are based on the mean estimate of the c coefficient for the improved TMP approach, while error bars reflect the respective 95% confidence interval corresponding to the linear fit of c (n = 8 five years estimates).

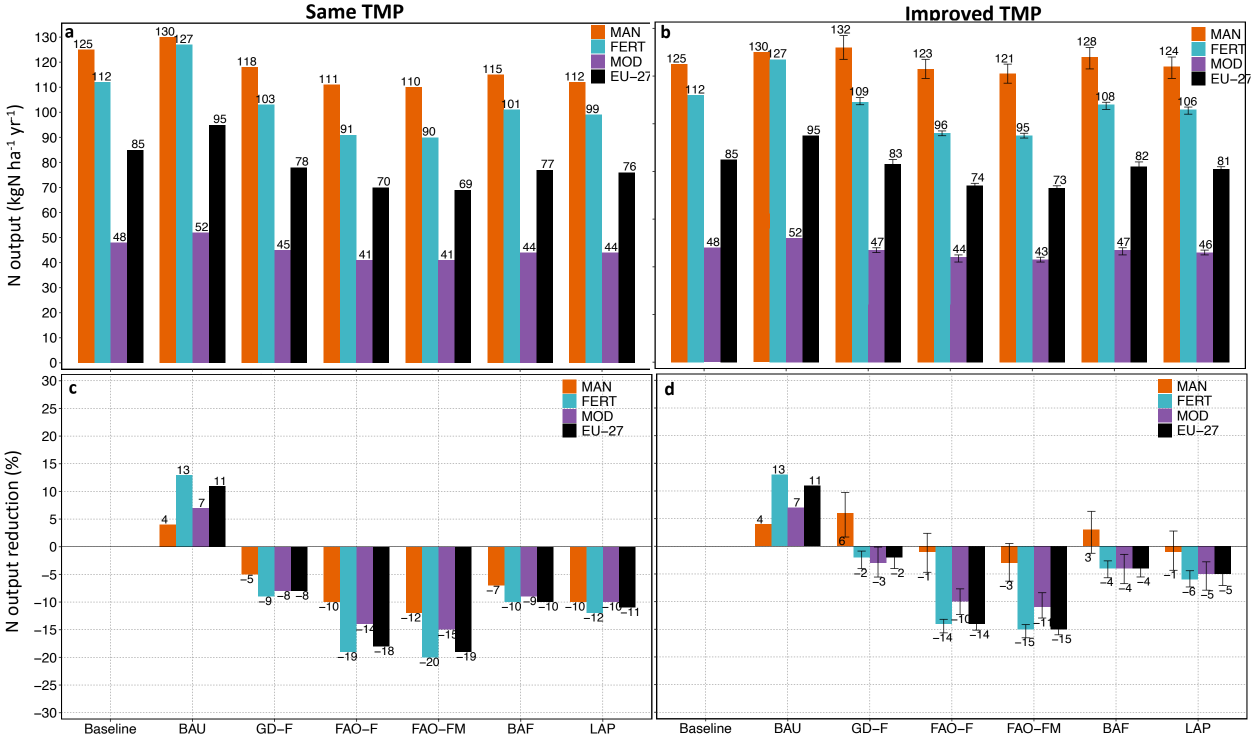

Figure S3: **Cropland N output projections for different typologies and EU-27 by 2030 under different intervention scenarios**. **(a,b)** Cropland N output (kgN ha^{-1} of cropland area yr^{-1}) in baseline, business-as-usual (BAU), and under specific scenarios using the same TMP and the improved TMP approaches, respectively. Results corresponding to the specific five N surplus reduction scenarios are represented by points in the panels (a,b) as: GD-F (Green Deal Fertilizer), FAO-F (FAO Fertilizer), FAO-FM (FAO Fertilizer and Manure), BAF (Better Animal Feed), and LAP (Less Animal Product). **(c,d)** Cropland N output changes (%) in the BAU and specific scenarios relative to the baseline period estimates with the same TMP and the improved TMP approach, respectively. Values on the bars in panels (b) and (d) are based on the mean estimate of the c coefficient for the improved TMP approach, while error bars reflect the respective 95% confidence interval corresponding to the linear fit of c (n = 8 five years estimates).

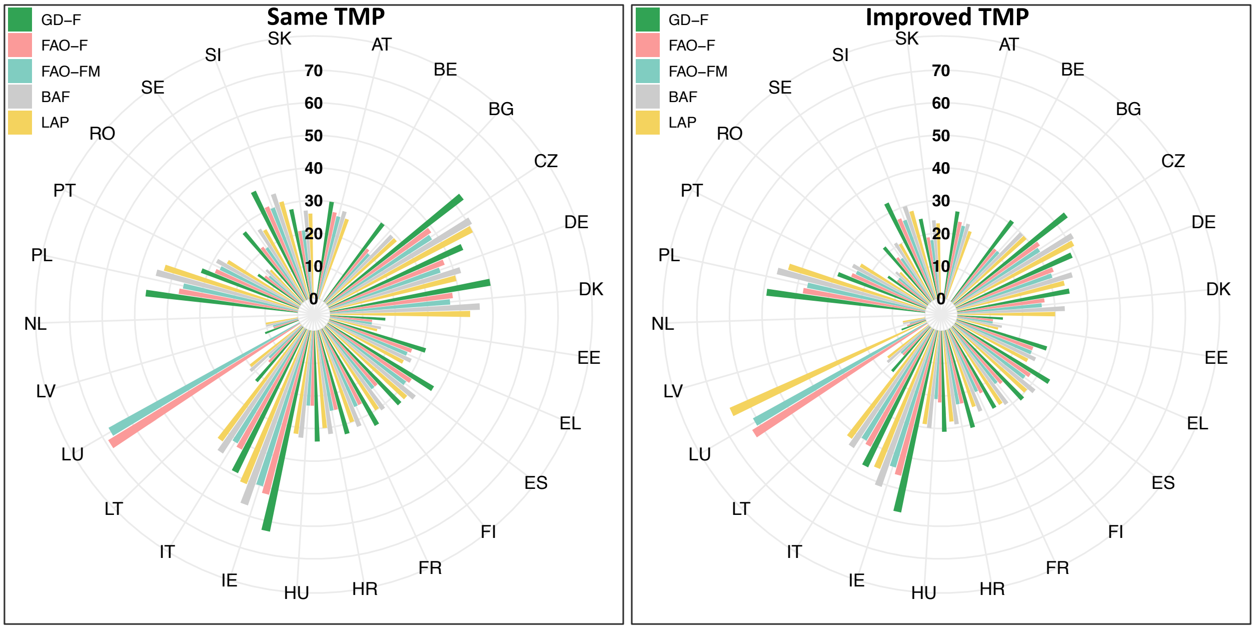

Figure S4: **N surplus (kgN ha^{-1} of agricultural area yr^{-1}) by 2030 compared to the baseline year (2015-2019) at country level under five future N surplus reduction scenarios using same and improved TMP approach.**

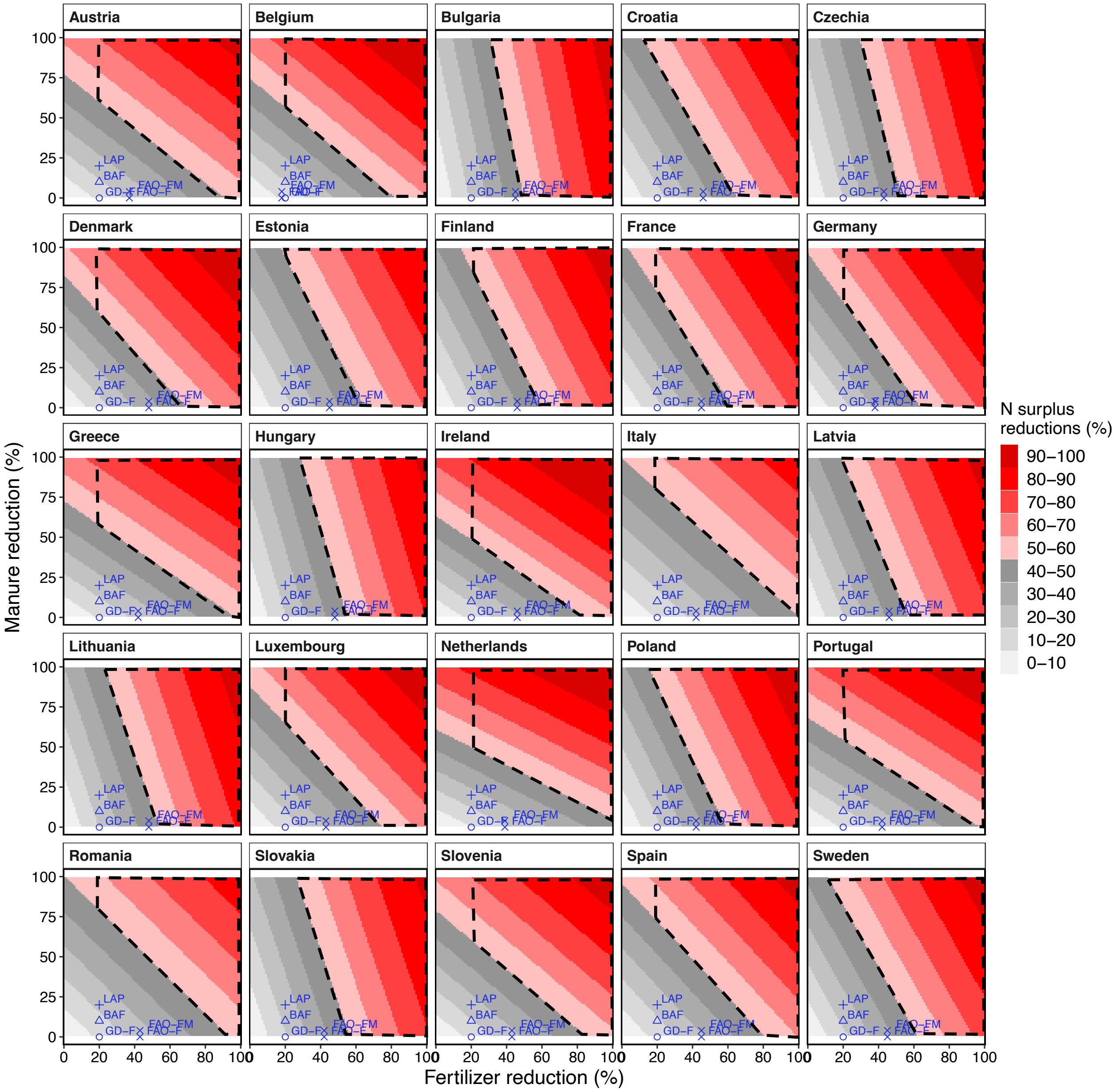

Figure S5: **N surplus reductions (%) compared to the baseline year (average between 2015 - 2019) depending on the mineral fertilizer and animal manure reduction and for different EU countries by 2030 under same TMP approach**. The selected five N surplus reduction scenarios are represented by points, including GD-F (Green Deal Fertilizer), FAO-F (FAO Fertilizer), FAO-FM (FAO Fertilizer and Manure), BAF (Better Animal Feed), and LAP (Less Animal Product). The dotted polygons represent the desired space where the target of 50% N surplus reduction is achieved while reducing mineral fertilizer by at least 20% according to the Green Deal F2F strategy.

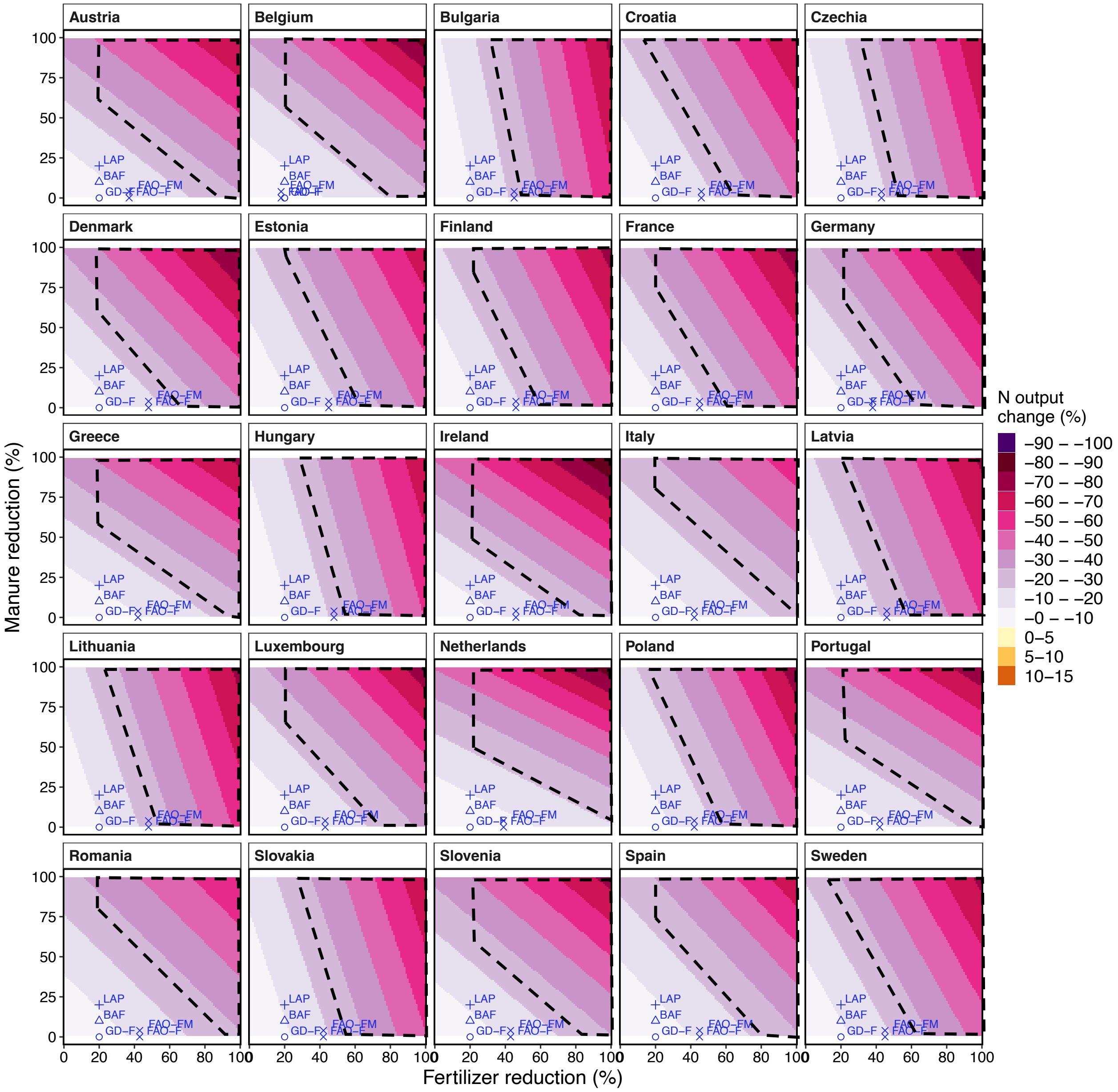

Figure S6: **N output change (%) compared to the baseline year (average between 2015 - 2019) depending on the mineral fertilizer and animal manure reduction and for different EU countries by 2030 under same TMP approach**. The selected five N surplus reduction scenarios are represented by points, including GD-F (Green Deal Fertilizer), FAO-F (FAO Fertilizer), FAO-FM (FAO Fertilizer and Manure), BAF (Better Animal Feed), and LAP (Less Animal Product). The dotted polygons represent the desired space where the target of 50% N surplus reduction is achieved while reducing mineral fertilizer by at least 20% according to the Green Deal F2F strategy.

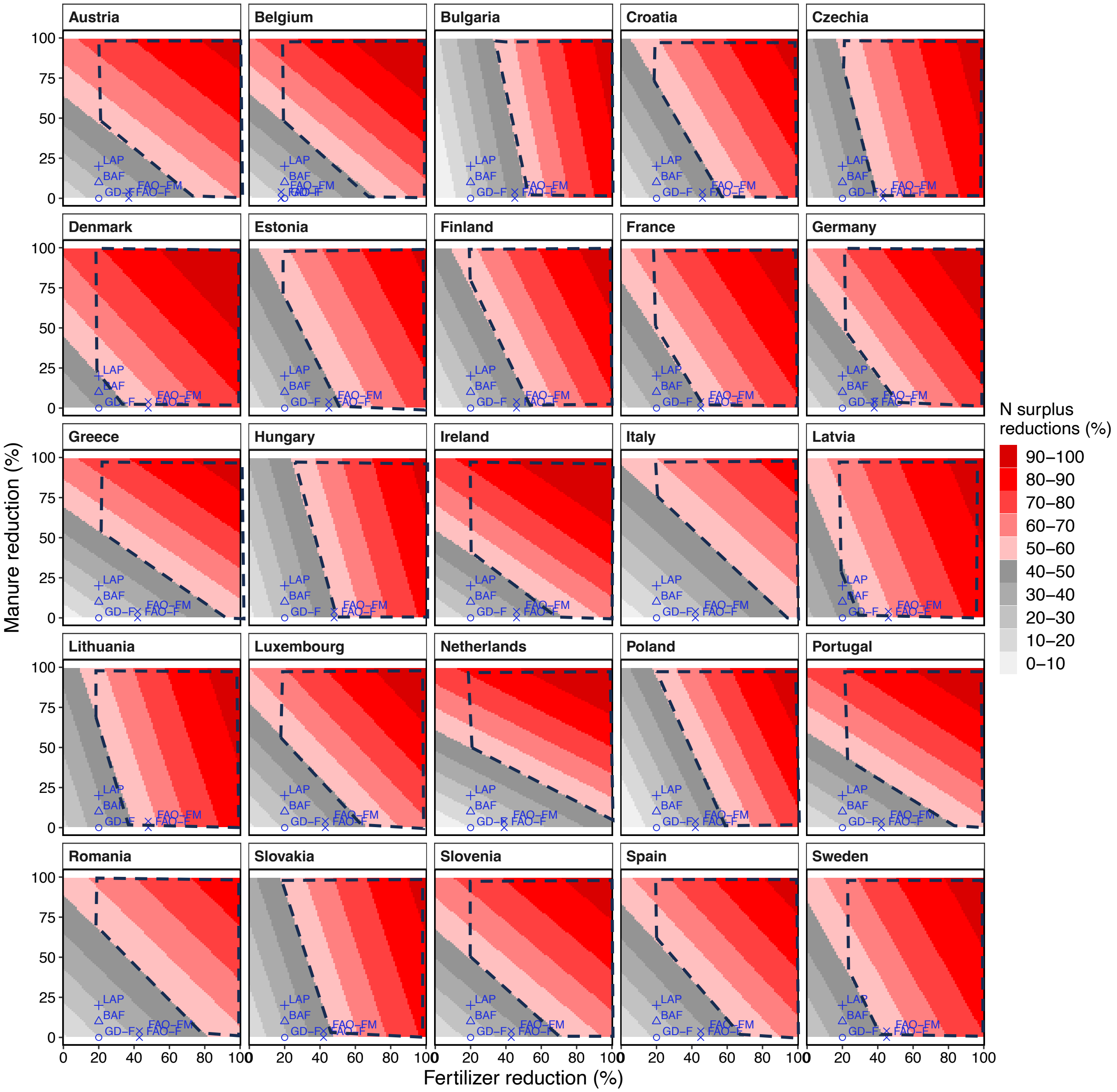

Figure S7: **N surplus reductions (%) compared to the baseline year (average between 2015 - 2019) depending on the mineral fertilizer and animal manure reduction and for different EU countries by 2030 under improved TMP approach**. The selected five N surplus reduction scenarios are represented by points, including GD-F (Green Deal Fertilizer), FAO-F (FAO Fertilizer), FAO-FM (FAO Fertilizer and Manure), BAF (Better Animal Feed), and LAP (Less Animal Product). The dotted polygons represent the desired space where the target of 50% N surplus reduction is achieved while reducing mineral fertilizer by at least 20% according to the Green Deal F2F strategy.

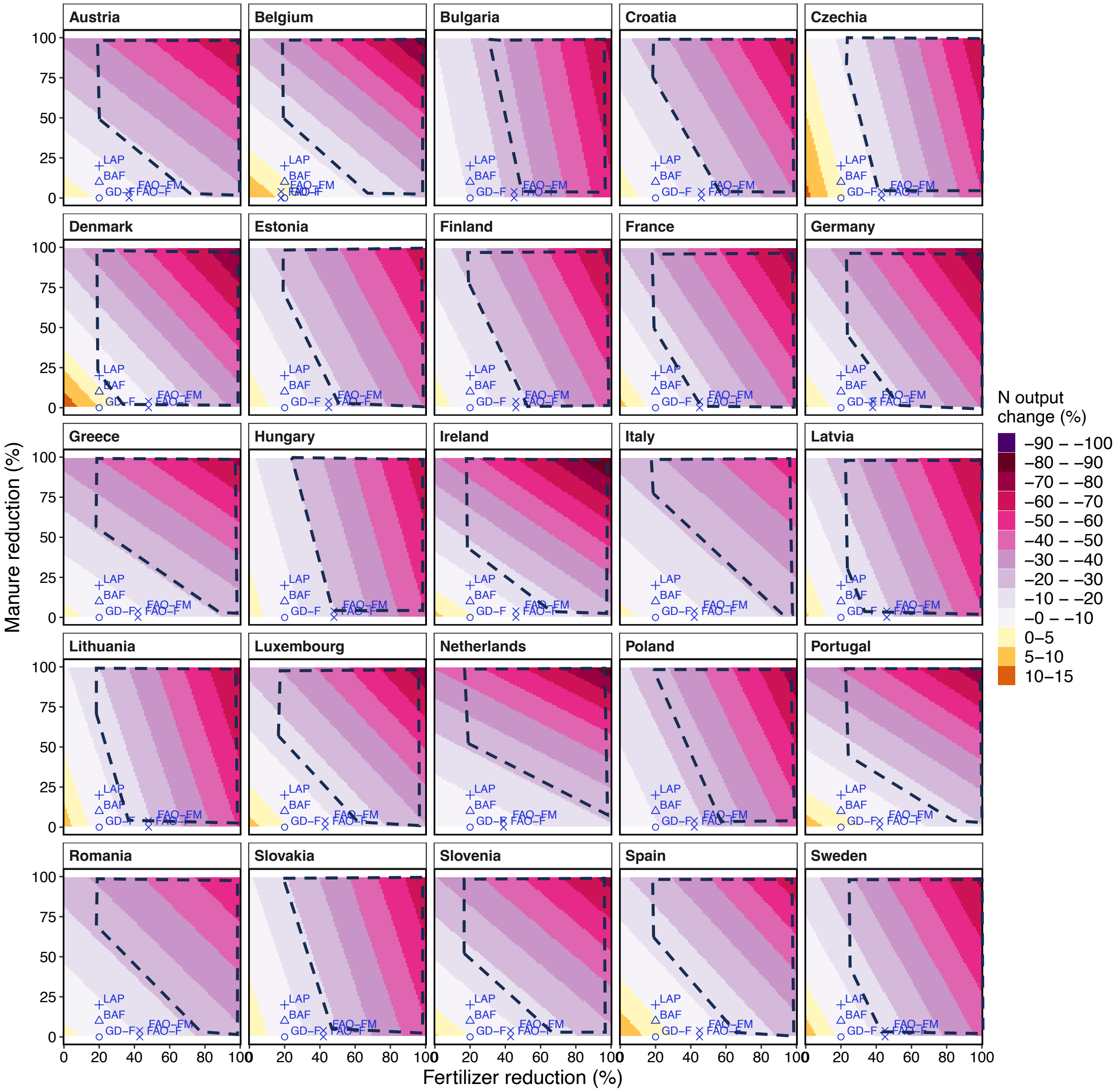

Figure S8: **N output change (%) compared to the baseline year (average between 2015 - 2019) depending on the mineral fertilizer and animal manure reduction and for different EU countries by 2030 under improved TMP approach**. The selected five N surplus reduction scenarios are represented by points, including GD-F (Green Deal Fertilizer), FAO-F (FAO Fertilizer), FAO-FM (FAO Fertilizer and Manure), BAF (Better Animal Feed), and LAP (Less Animal Product). The dotted polygons represent the desired space where the target of 50% N surplus reduction is achieved while reducing mineral fertilizer by at least 20% according to the Green Deal F2F strategy.

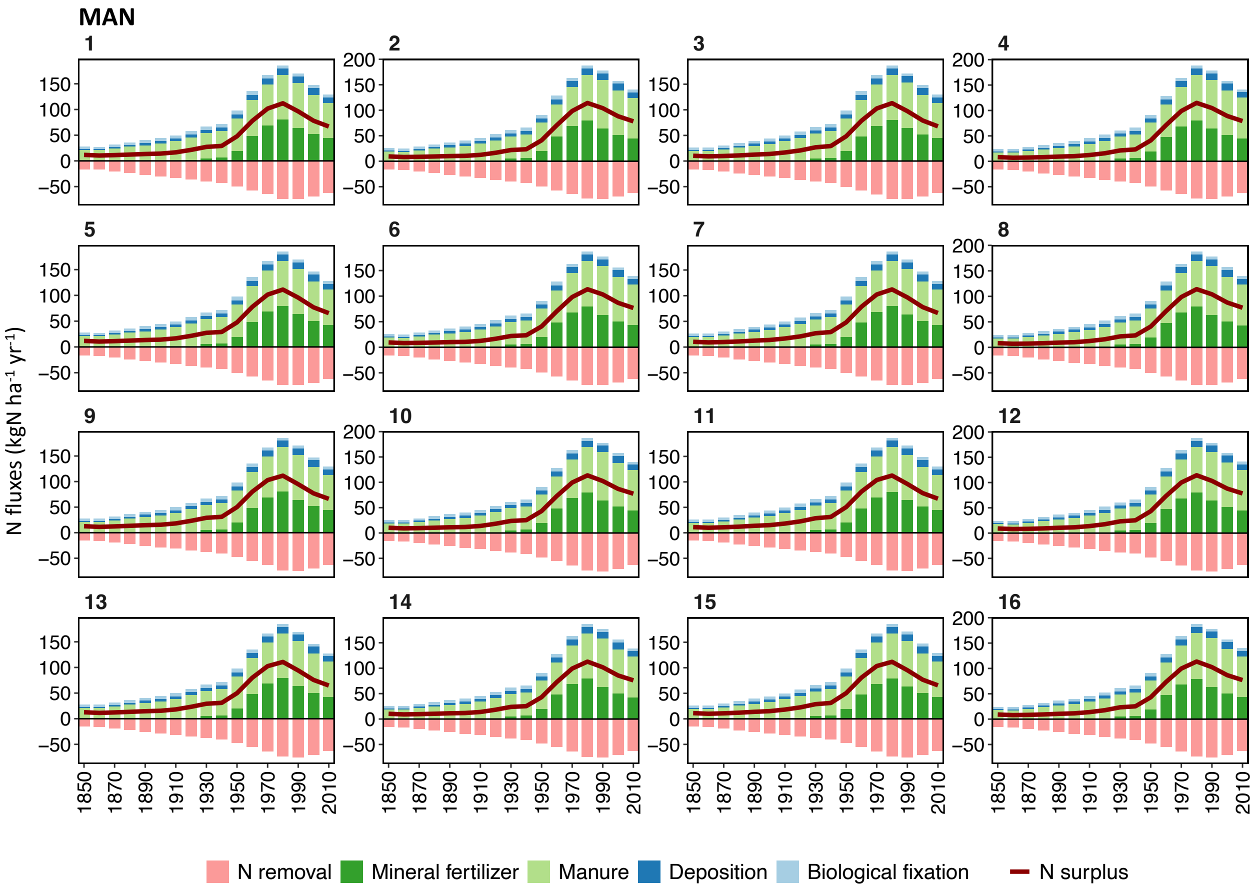

Figure S9: **Long-term time series of of total 16 N surplus (kgN ha^{-1} of total area yr^{-1}) and its components in MAN cluster over the period 1850-2019**. The bars indicate decadal mean values for the different N surplus components.

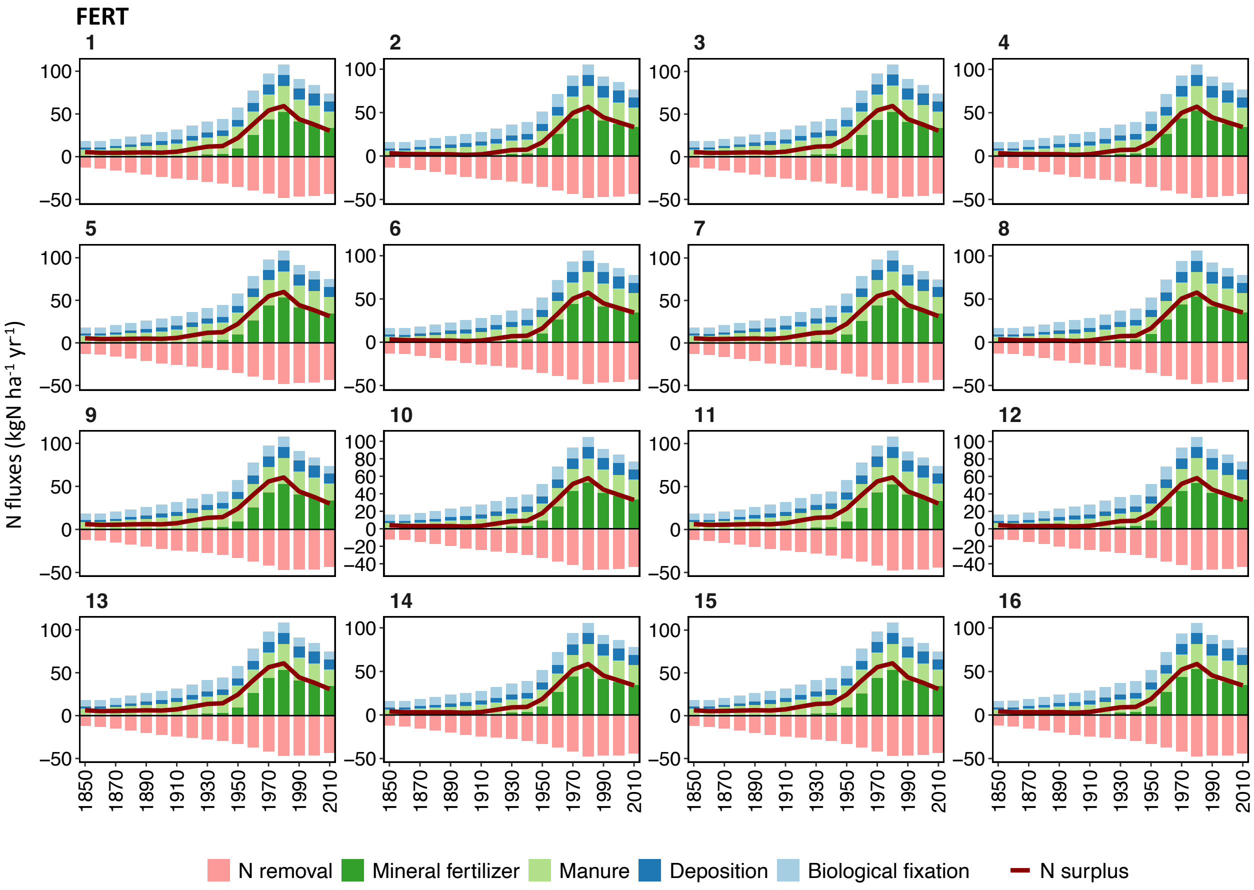

Figure S10: **Long-term time series of total 16 N surplus (kgN ha^{-1} of total area yr^{-1}) and its components in FERT cluster over the period 1850-2019**. The bars indicate decadal mean values for the different N surplus components.

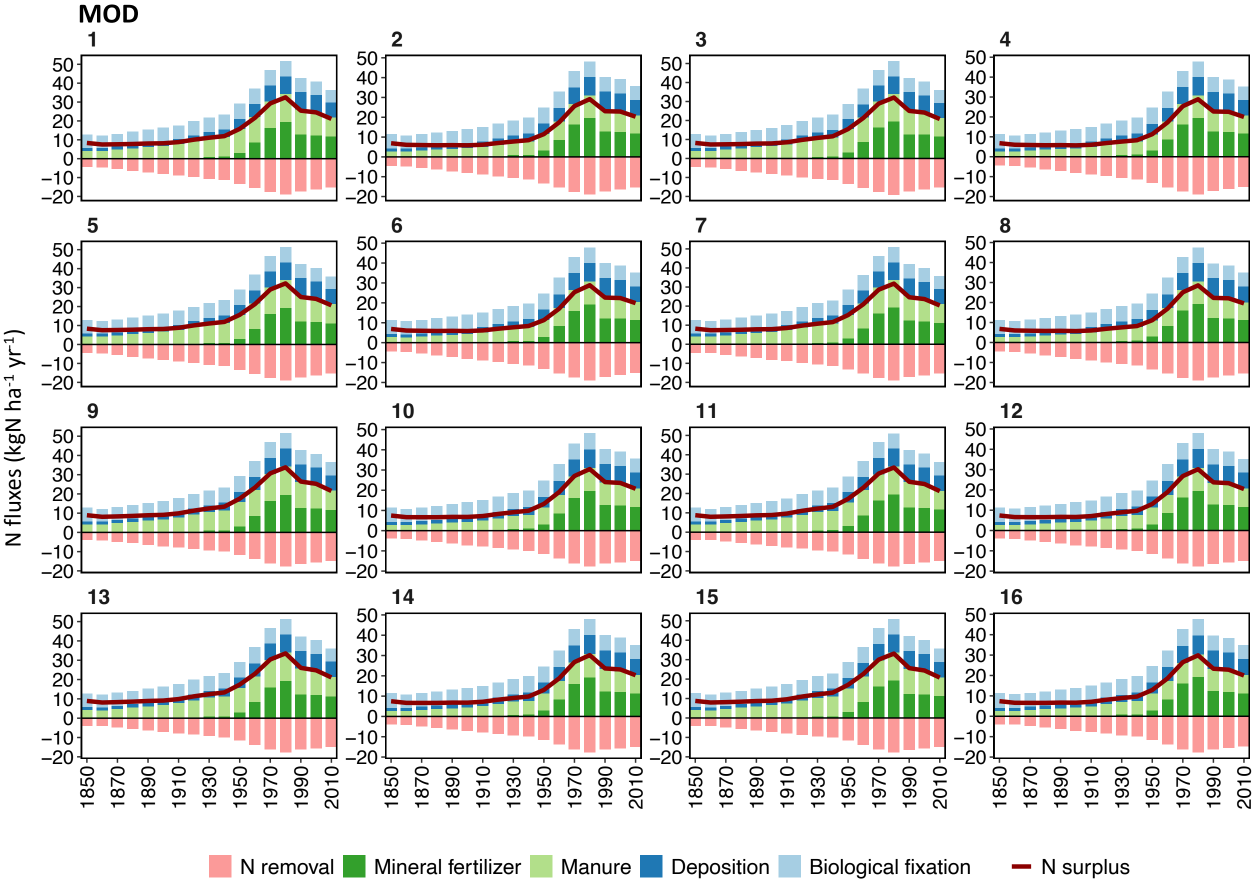

Figure S11: **Long-term time series of total 16 N surplus (kgN ha^{-1} of total area yr^{-1}) and its components in MOD cluster over the period 1850-2019**. The bars indicate decadal mean values for the different N surplus components.

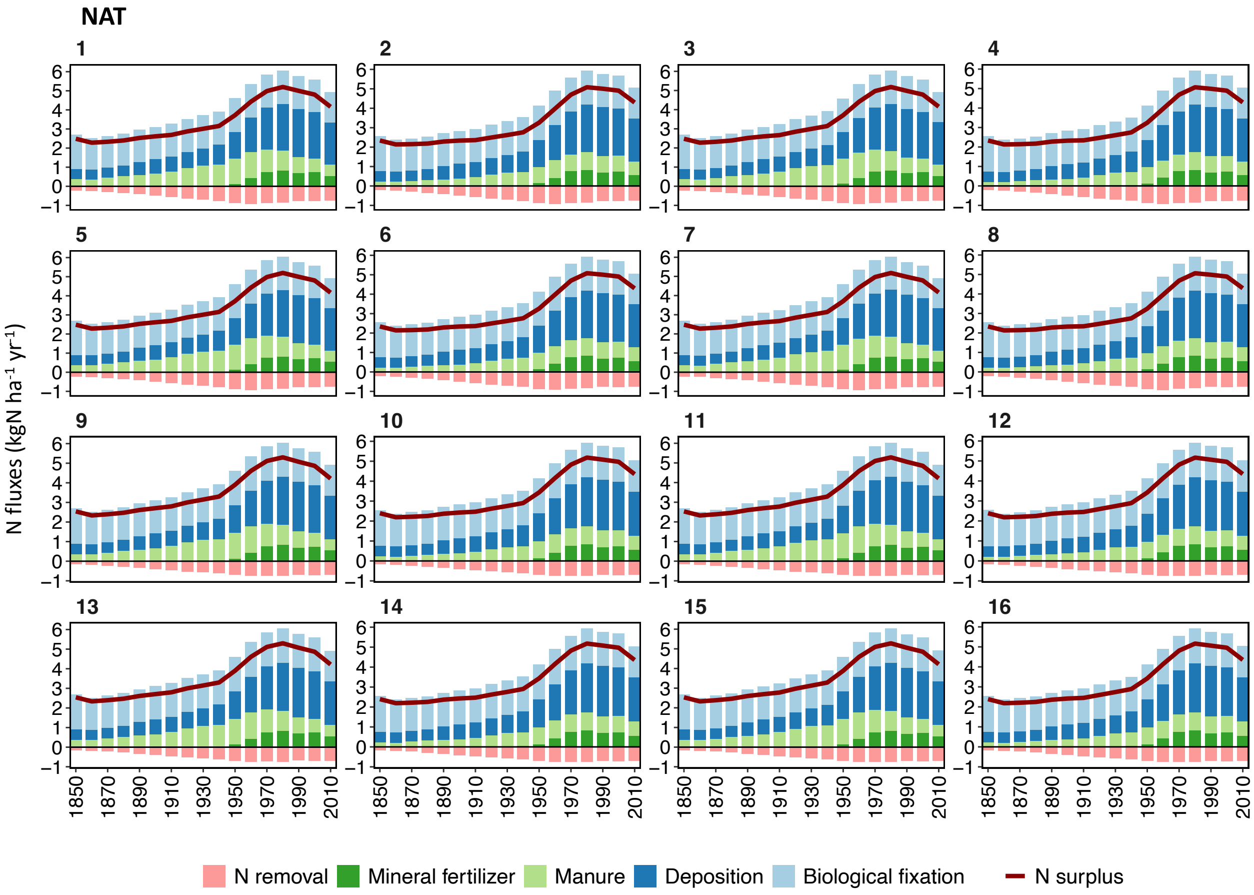

Figure S12: **Long-term time series of total 16 N surplus (kgN ha^{-1} of total area yr^{-1}) and its components in NAT cluster over the period 1850-2019**. The bars indicate decadal mean values for the different N surplus components.

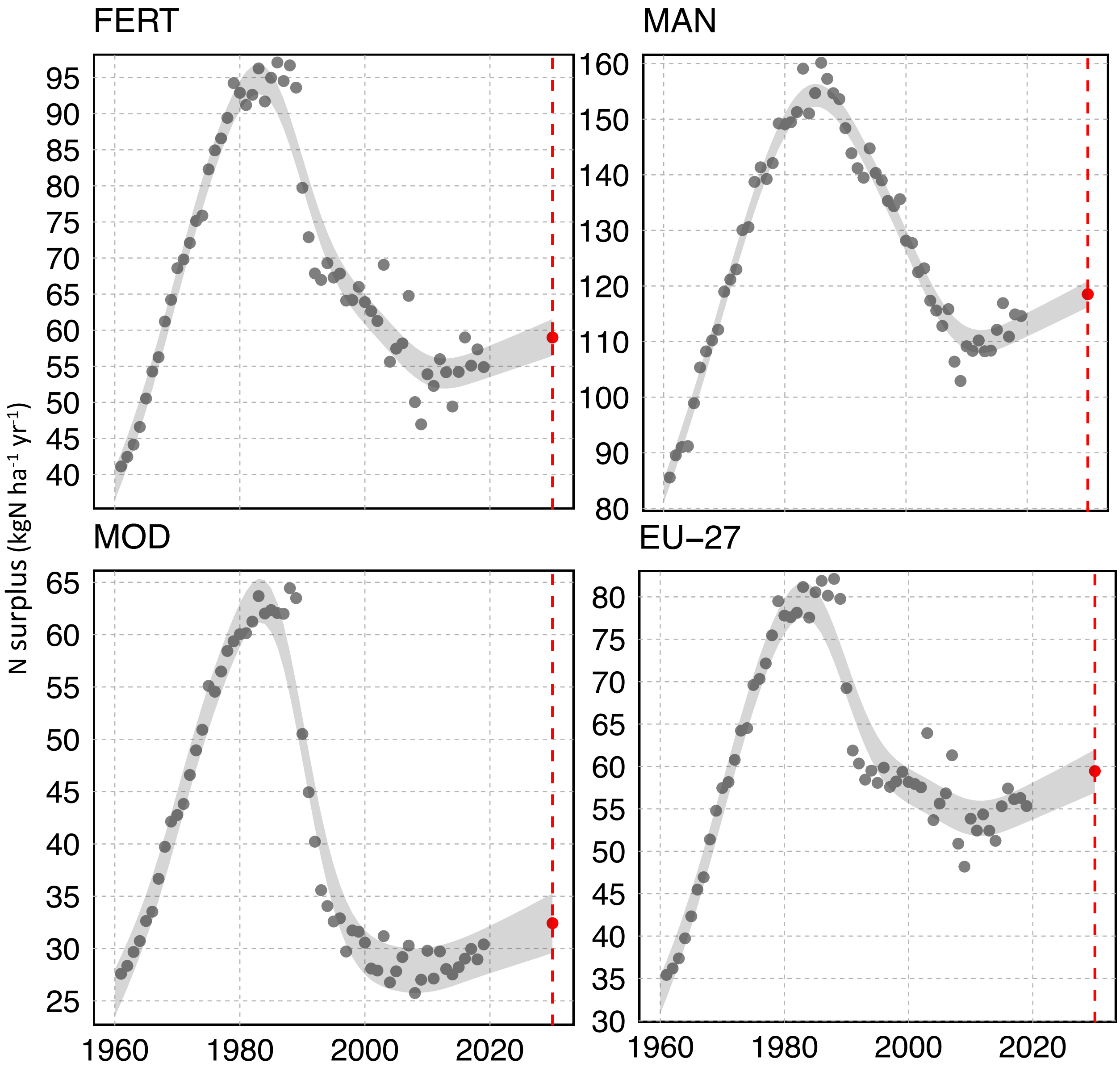

Figure S13: **Generalized additive model (GAM) fitting of N surplus (kgN ha^{-1} yr^{-1}) for clusters: FERT, MAN, MOD, and EU27, from 1961 to 2019.** The gray points show the annual estimates of N surplus for each typology and EU-27. The red points indicate the predicted values for 2030, while the grey shaded areas represent the 95% confidence intervals, illustrating the range of uncertainty in the model's predictions. The red dashed line marks the year 2030.

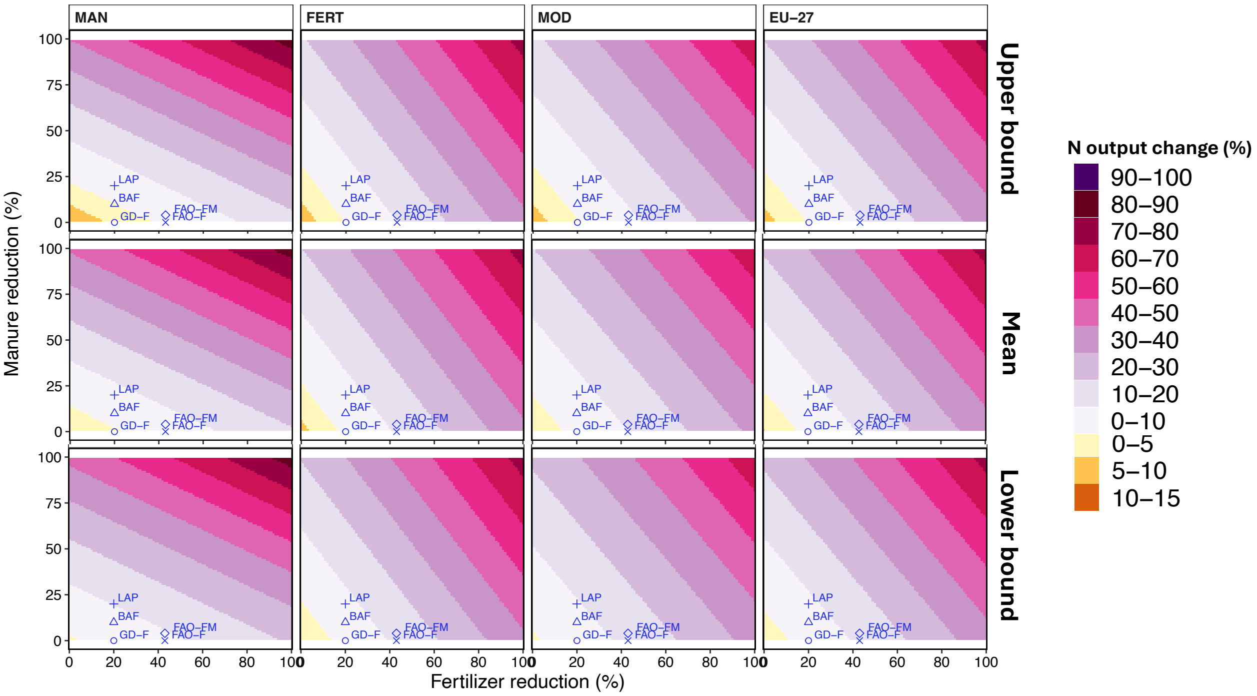

Figure S14: **Projected agricultural nitrogen (N) output changes (%) for different typologies and the EU-27 by 2030 under improved technological and management practices (TMPs).** The panels show the percentage change in N output relative to the baseline period (2015–2019) across a full range of scenarios (bottom-up analysis), considering different combinations of fertilizer and animal manure reductions under the improved TMPs approach. Each row represents a different assumption on the coefficient values (c) (see Eq. 4 in the main text)) in the one-parameter hyperbolic function: the top row uses the upper bound of the uncertainty range, the bottom row uses the lower bound, and the middle row shows projections based on the central (predicted) value of c. The uncertainty in c was derived by fitting a linear model to historical values (1981–2019) and calculating a 95% confidence interval. Results for the five selected N surplus reduction scenarios (top-down approach) are indicated as individual points in each panel as: GD-F (Green Deal Fertilizer), FAO-F (FAO Fertilizer), FAO-FM (FAO Fertilizer and Manure), BAF (Better Animal Feed), and LAP (Less Animal Product).

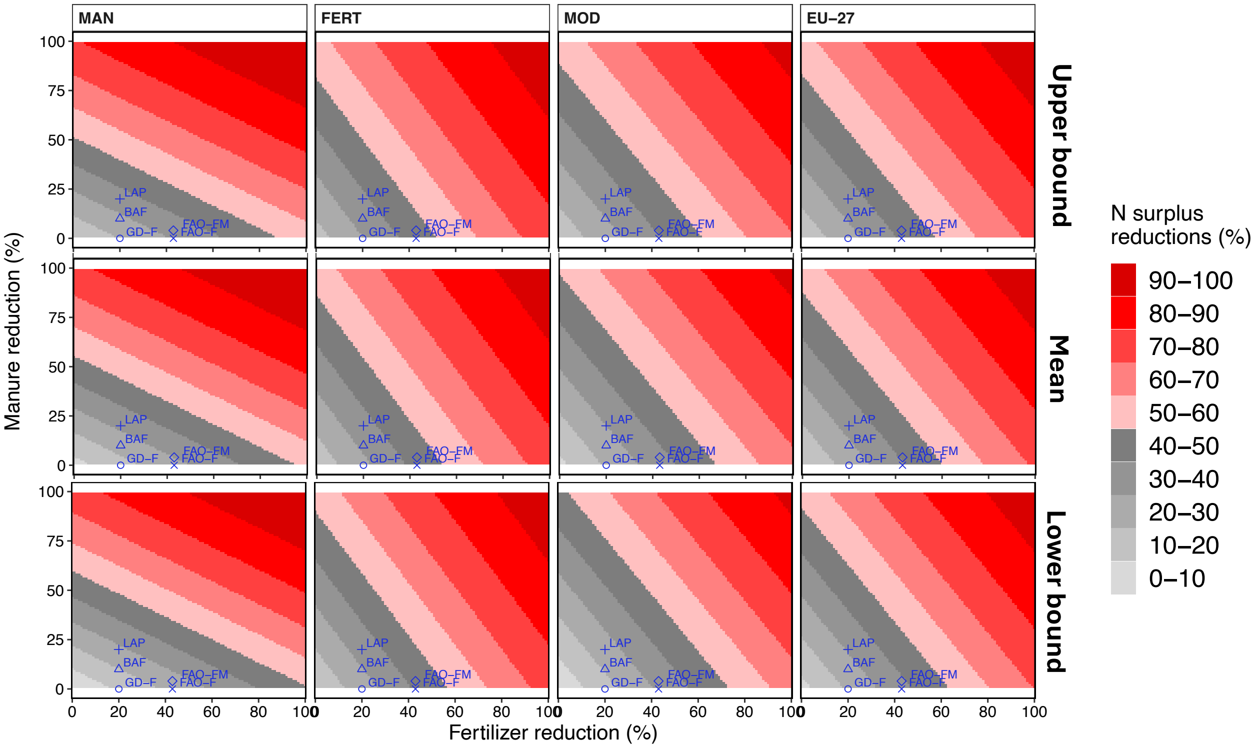

Figure S15: **Projected agricultural nitrogen (N) surplus changes (%) for different typologies and the EU-27 by 2030 under improved technological and management practices (TMPs).** The panels show the percentage change in N surplus relative to the baseline period (2015–2019) across a full range of scenarios (bottom-up analysis), considering different combinations of fertilizer and animal manure reductions under the improved TMPs approach. Each row represents a different assumption on the coefficient values (c) (see Eq. 4 in the main text)) in the one-parameter hyperbolic function: the top row uses the upper bound of the uncertainty range, the bottom row uses the lower bound, and the middle row shows projections based on the central (predicted) value of c. The uncertainty in c was derived by fitting a linear model to historical values (1981–2019) and calculating a 95% confidence interval. Results for the five selected N surplus reduction scenarios (top-down approach) are indicated as individual points in each panel as: GD-F (Green Deal Fertilizer), FAO-F (FAO Fertilizer), FAO-FM (FAO Fertilizer and Manure), BAF (Better Animal Feed), and LAP (Less Animal Product).

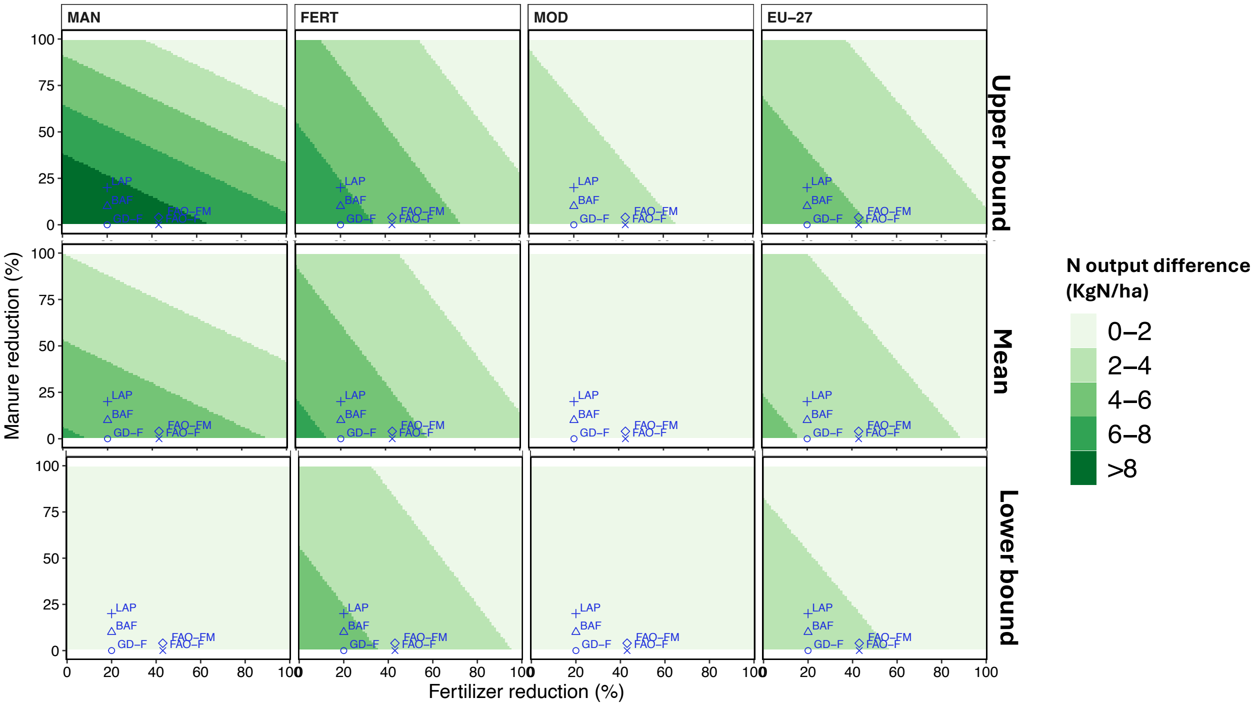

Figure S16: **Projected difference in agricultural nitrogen (N) output (kgN ha^{-1} of agricultural area yr^{-1}) between improved and same technological and management practices (TMPs) for different typologies and the EU-27 by 2030.** The panels show the absolute difference in projected N output between improved TMP and same TMP approaches across a full range of fertilizer and animal manure reduction scenarios (bottom-up analysis). Each row reflects a different assumption about the future value in the coefficient values (c) used in the one-parameter hyperbolic function: the top row uses the upper bound of the uncertainty range, the bottom row uses the lower bound, and the middle row shows projections based on the central (predicted) value of c. Uncertainty in c was derived from a linear model fitted to historical values (1981–2019), using a 95% confidence interval. Scenario-specific points represent the five selected N surplus reduction scenarios: GD-F (Green Deal Fertilizer), FAO-F (FAO Fertilizer), FAO-FM (FAO Fertilizer and Manure), BAF (Better Animal Feed), and LAP (Less Animal Product).

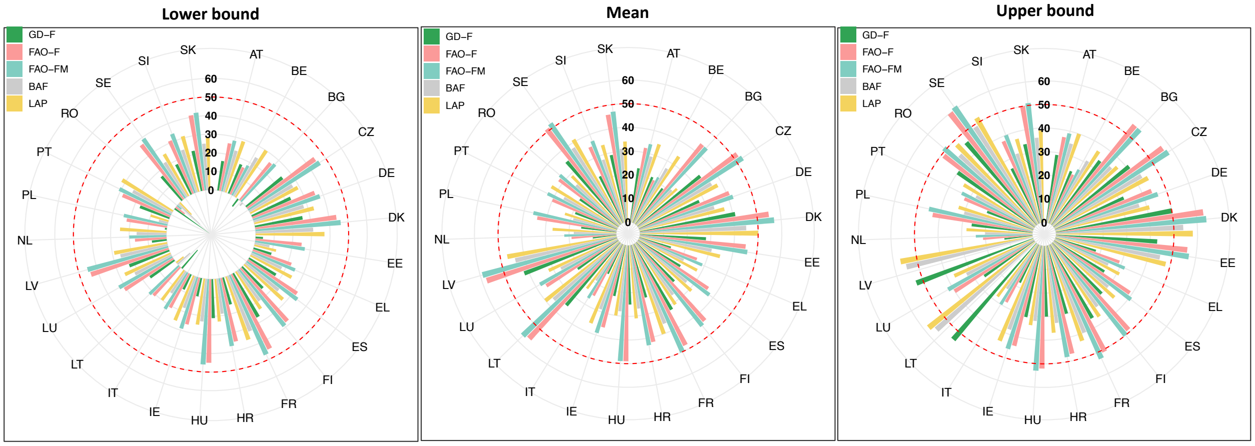

Figure S17: **Projected agricultural nitrogen surplus reduction (%) by 2030 relative to baseline estimates (2015–2019) for the EU-27 countries.** Each panel displays the lower bound, mean estimate, and upper bound of the 95% confidence interval, respectively, based on uncertainty in the coefficient values (c) (see Eq. 4 in the main text). These values were derived by fitting a one-parameter hyperbolic function using a linear model to historical data (1981–2019), and calculating the corresponding confidence interval. Results are shown for both the same TMP and improved TMP approaches across five scenarios: GD-F (Green Deal Fertilizer), FAO-F (FAO Fertilizer), FAO-FM (FAO Fertilizer and Manure), BAF (Better Animal Feed), and LAP (Less Animal Product). The red dashed circle indicates the EU Green Deal Farm to Fork strategy target of a 50% reduction in nitrogen surplus by 2030.

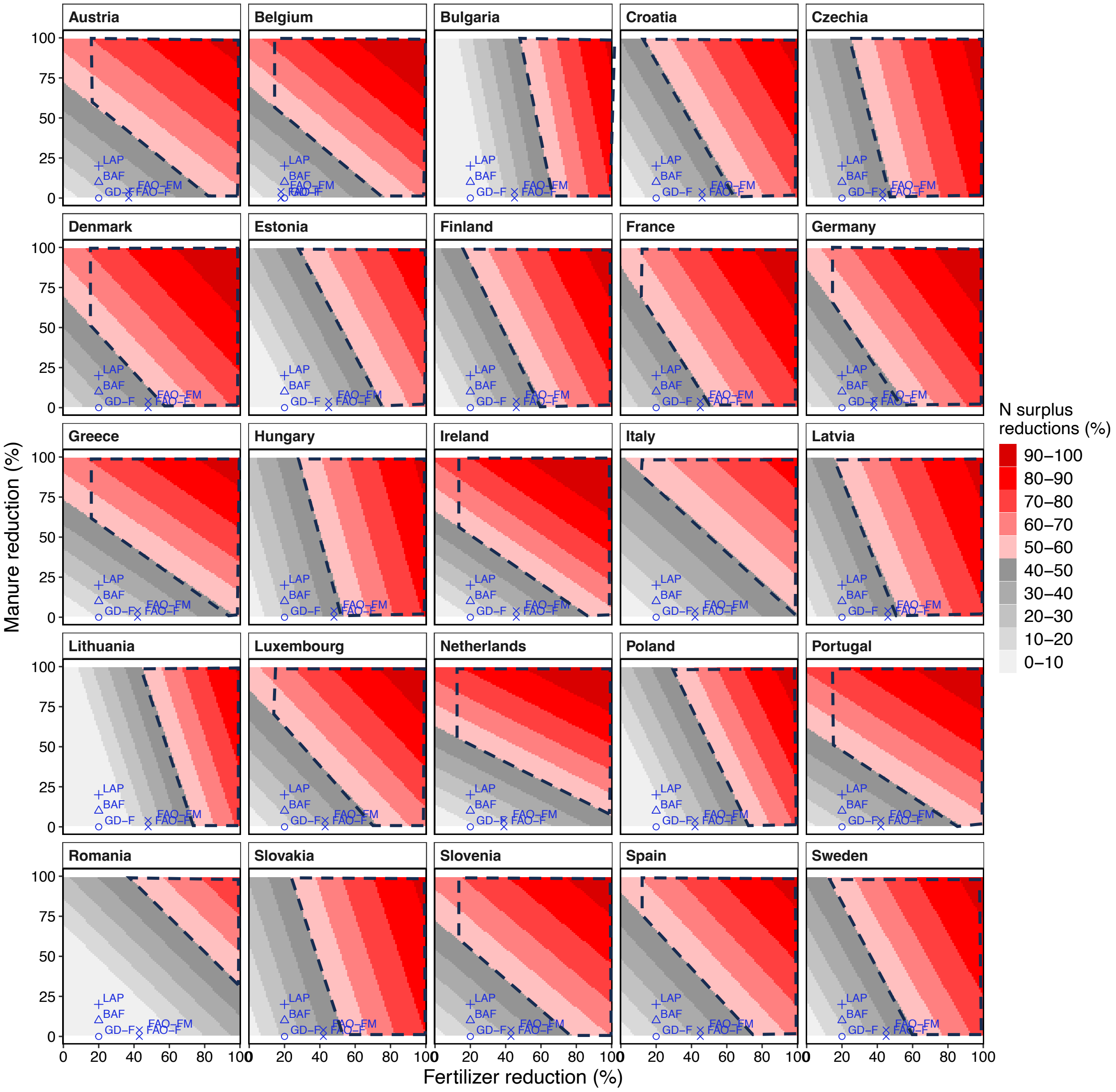

Figure S18: **Lower bound based projected agricultural nitrogen surplus reduction (%) by 2030 relative to baseline estimates (2015–2019) for the EU-27 countries**. Values are based on estimates using the lower limit of the 95% confidence interval for the yield response coefficient (c), derived from a linear model fit to historical data (1981–2019).

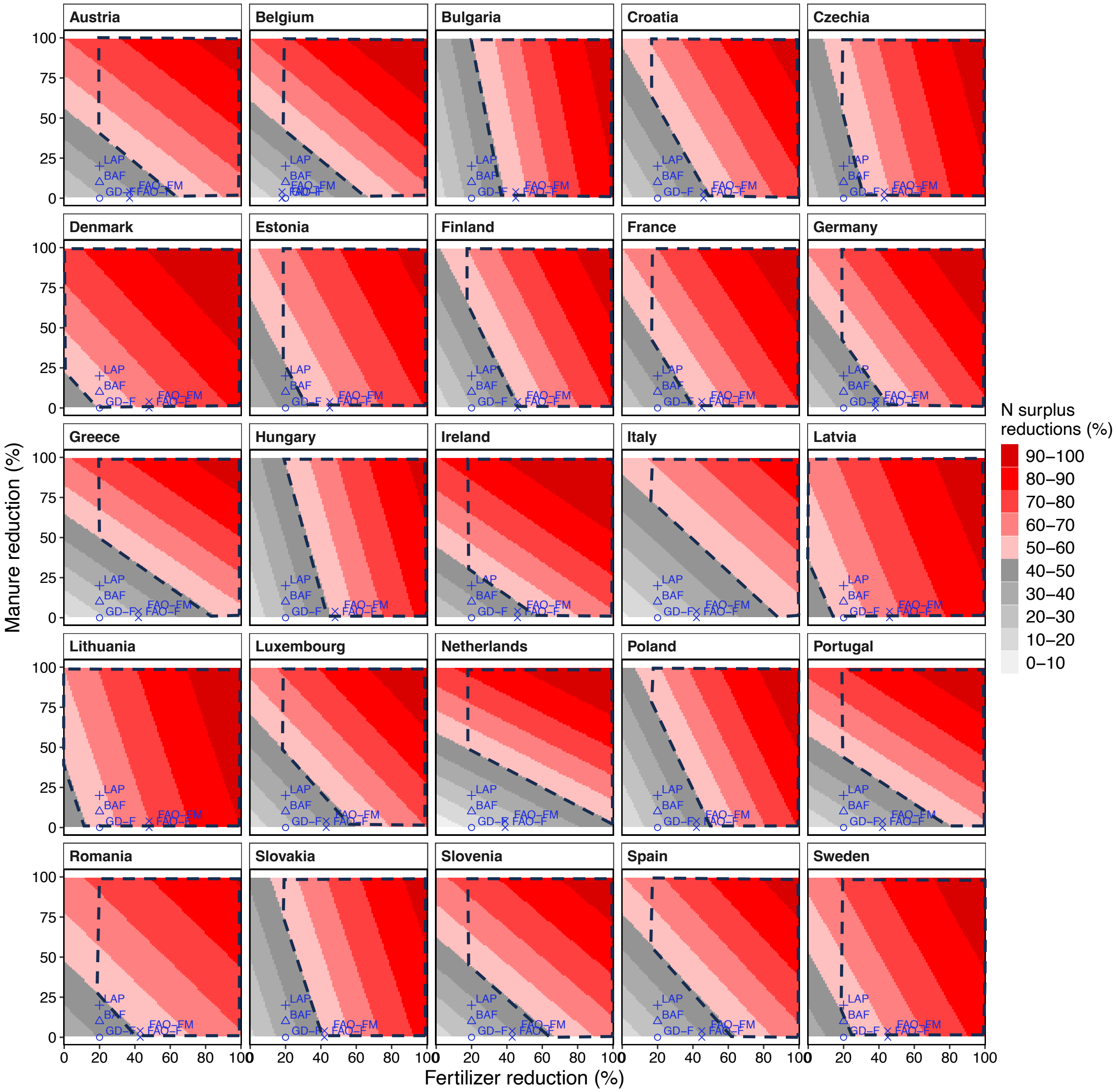

Figure S19: **Upper bound based projected agricultural nitrogen surplus reduction (%) by 2030 relative to baseline estimates (2015–2019) for the EU-27 countries**. Values are based on estimates using the upper limit of the 95% confidence interval for the yield response coefficient (c), derived from a linear model fit to historical data (1981–2019).

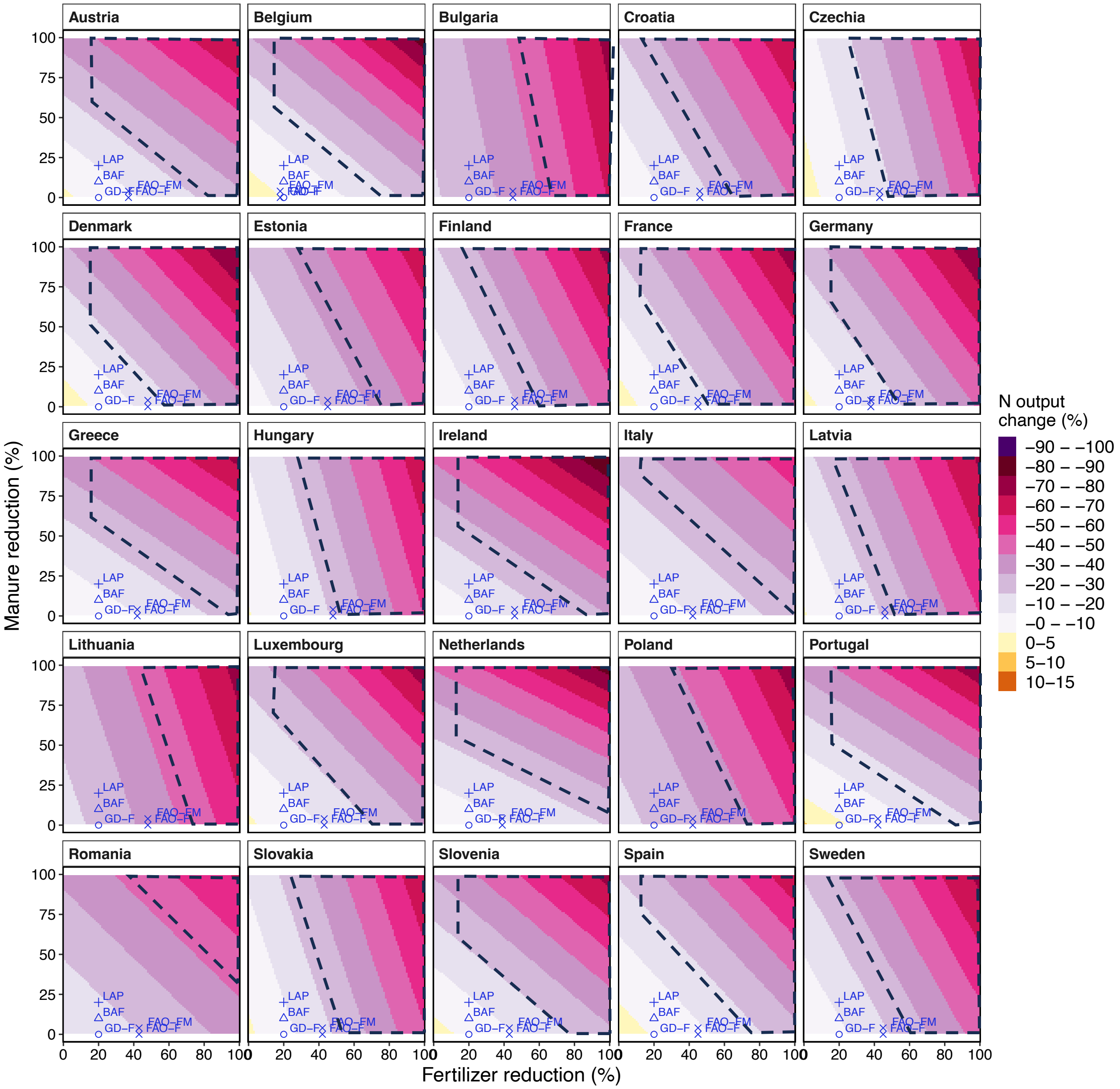

Figure S20: **Lower bound based projected agricultural nitrogen output change (%) by 2030 relative to baseline estimates (2015–2019) for the EU-27 countries.** Values are based on estimates using the lower limit of the 95% confidence interval for the yield response coefficient (c), derived from a linear model fit to historical data (1981–2019).

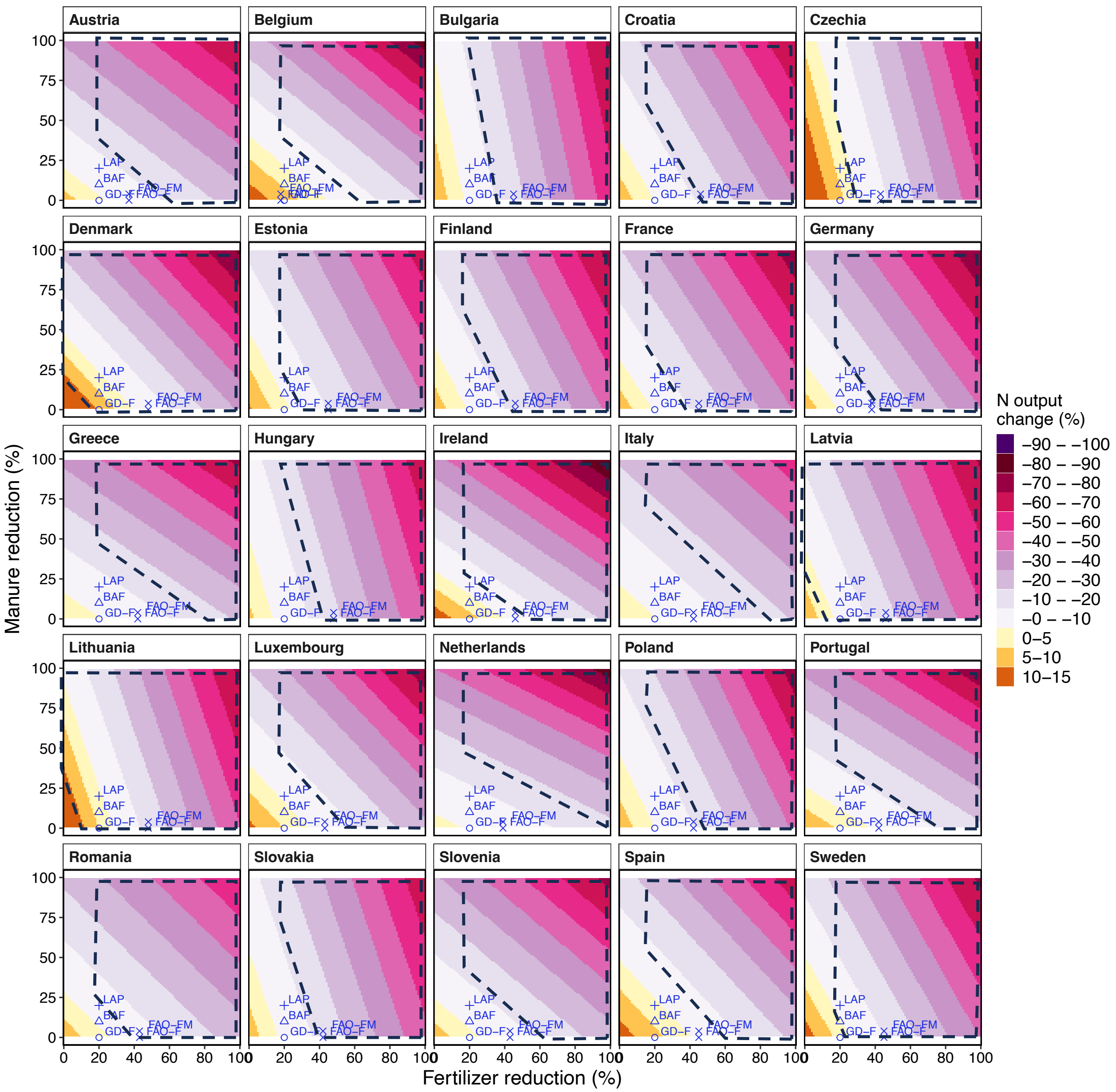

Figure S21: **Upper bound based projected agricultural nitrogen output change (%) by 2030 relative to baseline estimates (2015–2019) for the EU-27 countries.** Values are based on estimates using the upper limit of the 95% confidence interval for the yield response coefficient (c), derived from a linear model fit to historical data (1981–2019).

Table S1: Projected values for the coefficient c (kgN ha^{-1} yr^{-1}) in 2030 (see Eq. 4 in the main text), obtained from a linear fit of the one-parameter hyperbolic functions for different Nitrogen surplus typologies and the EU-27 level. Results are shown for both the same and improved TMP approaches. For the improved TMP, the table reports the fitted value (Mean) along with its 95% confidence interval (Lower and Upper bounds), which represent the statistical uncertainty associated with the parameter estimate. See the Methods section for further details.

**Typologies**	**Same TMP**	**Improved TMP**		
		**Lower**	**Mean**	**Upper**
MAN	279	285	313	341
FERT	304	357	373	389
MOD	122	126	138	150
EU-27	229	255	267	279

1. FAOSTAT (Food and Agriculture Organization Corporate Statistical Database): Fertilizer by nutrients, available at:. `https://www.fao.org/faostat/en/#data/RFN`. [Accessed: 10-October-2021].

2. Holland, E., Lee-Taylor, J., Nevison, C. & Sulzman, J. Global n cycle: Fluxes and n2o mixing ratios originating from human activity. *ORNL DAAC* (2005).

3. Pollock, D. Nitrates, water and salt: maintaining the fertility of agriculture. *Interdisciplinary Science Reviews* **32**, 350–360 (2007).

4. Heffer, P., Gruère, A., Roberts, T. *et al.* Assessment of fertilizer use by crop at the global level. *International Fertilizer Industry Association, Paris* (2013).

5. FAOSTAT (Food and Agriculture Organization Corporate Statistical Database): Land Use domain, available at:. `https://www.fao.org/faostat/en/#data/RL/`. [Accessed: 10-August-2021].

6. Monfreda, C., Ramankutty, N. & Foley, J. A. Farming the planet: 2. geographic distribution of crop areas, yields, physiological types, and net primary production in the year 2000. *Global biogeochemical cycles* **22** (2008).

7. Einarsson, R. *et al.* Crop production and nitrogen use in european cropland and grassland 1961–2019. *Scientific Data* **8**, 288 (2021).

8. FAOSTAT (Food and Agriculture Organization Corporate Statistical Database): Livestock Manure, available at:. `https://www.fao.org/faostat/en/#data/EMN`. [Accessed: 10-October-2021].

9. Dong, H. *et al.* 2006 IPCC Guidelines for National Greenhouse Gas inventories - Chapter 10: Emissions from livestock and manure management. *Report* (2020).

10. Lassaletta, L., Billen, G., Grizzetti, B., Anglade, J. & Garnier, J. 50 year trends in nitrogen use efficiency of world cropping systems: the relationship between yield and nitrogen input to cropland. *Environmental Research Letters* **9**, 105011 (2014).

11. Zhang, B. *et al.* Global manure nitrogen production and application in cropland during 1860–2014: a 5 arcmin gridded global dataset for earth system modeling. *Earth System Science Data* **9**, 667–678 (2017).

12. Robinson, T. P. *et al.* Mapping the global distribution of livestock. *PloS one* **9**, e96084 (2014).

13. Tian, H. *et al.* The global n2o model intercomparison project. *Bulletin of the American Meteorological Society* **99**, 1231–1251 (2018).

14. input4mips (input datasets for Model Intercomparison Projects): N deposition, available at:. `https://esgf-node.llnl.gov/search/input4mips/`. [Accessed: 10-July-2021].

15. Batool, M. *et al.* Long-term annual soil nitrogen surplus across europe (1850–2019). *Scientific Data* **9**, 612 (2022).
