# Supplementary material for: Scenario analysis of nitrogen surplus typologies in Europe shows that a 20% fertilizer reduction may fall short of 2030 EU Green Deal goals
Source: Nat Food. 2025 Aug 19;6(8):787–98. doi: 10.1038/s43016-025-01210-2 (PMC12367536; doi:10.1038/s43016-025-01210-2)
Supplement: Supplementary file 2 — Reporting Summary [file 43016_2025_1210_MOESM2_ESM.pdf]

nature portfolio

Corresponding author(s): Masooma Batool
Rohini Kumar

Last updated by author(s): Jun 10, 2025

Reporting Summary

Nature Portfolio wishes to improve the reproducibility of the work that we publish. This form provides structure for consistency and transparency in reporting. For further information on Nature Portfolio policies, see our Editorial Policies and the Editorial Policy Checklist.

Statistics

For all statistical analyses, confirm that the following items are present in the figure legend, table legend, main text, or Methods section.

n/a	Confirmed	
☒	☐	The exact sample size (*n*) for each experimental group/condition, given as a discrete number and unit of measurement
☒	☐	A statement on whether measurements were taken from distinct samples or whether the same sample was measured repeatedly
☐	☒	The statistical test(s) used AND whether they are one- or two-sided<br>*Only common tests should be described solely by name; describe more complex techniques in the Methods section.*
☐	☒	A description of all covariates tested
☒	☐	A description of any assumptions or corrections, such as tests of normality and adjustment for multiple comparisons
☐	☒	A full description of the statistical parameters including central tendency (e.g. means) or other basic estimates (e.g. regression coefficient) AND variation (e.g. standard deviation) or associated estimates of uncertainty (e.g. confidence intervals)
☒	☐	For null hypothesis testing, the test statistic (e.g. *F*, *t*, *r*) with confidence intervals, effect sizes, degrees of freedom and *P* value noted<br>*Give P values as exact values whenever suitable.*
☒	☐	For Bayesian analysis, information on the choice of priors and Markov chain Monte Carlo settings
☒	☐	For hierarchical and complex designs, identification of the appropriate level for tests and full reporting of outcomes
☒	☐	Estimates of effect sizes (e.g. Cohen's *d*, Pearson's *r*), indicating how they were calculated

Our web collection on statistics for biologists contains articles on many of the points above.

Software and code

Policy information about availability of computer code

Data collection	The underlying nitrogen (N) surplus datasets are archived at the Zenodo repository here: https://zenodo.org/records/6581441. Also, the related materials supporting the findings of this study are available in the Github repository at the following URL: https://github.com/batool-masooma/N_surplus_typologies_Europe
Data analysis	The code used for data processing, analysis, and figure generation is archived on Zenodo (https://doi.org/10.5281/zenodo.15520404) and is also available on GitHub at: https://github.com/batool-masooma/N_surplus_typologies_Europe. Instructions for reproducing the results are provided in the accompanying README files.

For manuscripts utilizing custom algorithms or software that are central to the research but not yet described in published literature, software must be made available to editors and reviewers. We strongly encourage code deposition in a community repository (e.g. GitHub). See the Nature Portfolio guidelines for submitting code & software for further information.

Data

Policy information about availability of data

All manuscripts must include a data availability statement. This statement should provide the following information, where applicable:

- Accession codes, unique identifiers, or web links for publicly available datasets
- A description of any restrictions on data availability
- For clinical datasets or third party data, please ensure that the statement adheres to our policy

The datasets used in this study are publicly available in archived Zenodo repositories. The primary dataset (version 1.0) used to generate Figures 1–5, along with supporting materials, is available at Zenodo (https://doi.org/10.5281/zenodo.15520404). This includes: (1) all input datasets in .xlsx format; (2) output plots in publication-quality .pdf format; and (3) README files describing variables, units, and structure. The underlying nitrogen surplus dataset is available separately at Zenodo (https://zenodo.org/records/6581441).

Human research participants

Policy information about studies involving human research participants and Sex and Gender in Research.

Reporting on sex and gender	*Use the terms sex (biological attribute) and gender (shaped by social and cultural circumstances) carefully in order to avoid confusing both terms. Indicate if findings apply to only one sex or gender; describe whether sex and gender were considered in study design whether sex and/or gender was determined based on self-reporting or assigned and methods used. Provide in the source data disaggregated sex and gender data where this information has been collected, and consent has been obtained for sharing of individual-level data; provide overall numbers in this Reporting Summary. Please state if this information has not been collected. Report sex- and gender-based analyses where performed, justify reasons for lack of sex- and gender-based analysis.*
Population characteristics	*Describe the covariate-relevant population characteristics of the human research participants (e.g. age, genotypic information, past and current diagnosis and treatment categories). If you filled out the behavioural & social sciences study design questions and have nothing to add here, write "See above."*
Recruitment	*Describe how participants were recruited. Outline any potential self-selection bias or other biases that may be present and how these are likely to impact results.*
Ethics oversight	*Identify the organization(s) that approved the study protocol.*

Note that full information on the approval of the study protocol must also be provided in the manuscript.

Field-specific reporting

Please select the one below that is the best fit for your research. If you are not sure, read the appropriate sections before making your selection.

☐ Life sciences ☐ Behavioural & social sciences ☒ Ecological, evolutionary & environmental sciences

For a reference copy of the document with all sections, see nature.com/documents/nr-reporting-summary-flat.pdf

Ecological, evolutionary & environmental sciences study design

All studies must disclose on these points even when the disclosure is negative.

Study description	Europe
Research sample	Nitrogen (N) surplus for both agricultural and non-agricultural soils across Europe at a 5 arcmin (approximately 10 km at the Equator) spatial resolution from 1850 to 2019
Sampling strategy	na
Data collection	The collected underlying dataset has been recorded at Zenedo repository (https://zenodo.org/records/6581441) with detailed explanation of methodology and all source codes supporting the reconstruction of N surplus is published here: https://www.nature.com/articles/s41597-022-01693-9. The source code supporting the finding of this study is is archived on Zenodo (https://doi.org/10.5281/zenodo.15520404) and is also available on GitHub at: https://github.com/batool-masooma/N_surplus_typologies_Europe
Timing and spatial scale	1850-2019 at annual time step at a spatial scale of 5 arcmin (approximately 10 km at the equator) and NUTS (Nomenclature of Territorial Units for Statistics) 2 level
Data exclusions	na

Reproducibility	All source code (programmed in RStudio version (2023.12.1+402)) used to analyse the dataset has been documented and is archived on Zenodo (https://doi.org/10.5281/zenodo.15520404) and is also available on GitHub at: https://github.com/batool-masooma/N_surplus_typologies_Europe, making our work reproducible. The reconstruction of the underlying N-surplus dataset was documented with detailed steps in our article 'Long-term annual soil nitrogen surplus across Europe (1850-2019)', published in Nature scientific data here: https://www.nature.com/articles/s41597-022-01693-9.
Randomization	na
Blinding	The underlying dataset has been provided in Zenodo with DOI of "10.5281/zenodo.6581441" and "10.5281/zenodo.15520404" as already been document above under "Reproducibility" section.

Did the study involve field work? ☐ Yes ☒ No

Reporting for specific materials, systems and methods

We require information from authors about some types of materials, experimental systems and methods used in many studies. Here, indicate whether each material, system or method listed is relevant to your study. If you are not sure if a list item applies to your research, read the appropriate section before selecting a response.

Materials & experimental systems

n/a	Involved in the study
☒	☐ Antibodies
☒	☐ Eukaryotic cell lines
☒	☐ Palaeontology and archaeology
☒	☐ Animals and other organisms
☒	☐ Clinical data
☒	☐ Dual use research of concern

Methods

n/a	Involved in the study
☒	☐ ChIP-seq
☒	☐ Flow cytometry
☒	☐ MRI-based neuroimaging
